# Supplementary material for: miR-596-3p suppresses brain metastasis of non-small cell lung cancer by modulating YAP1 and IL-8
Source: Cell Death Dis. 2022 Aug 12;13(8):699. doi: 10.1038/s41419-022-05062-7 (PMC9374706; doi:10.1038/s41419-022-05062-7)
Supplement: Supplementary file 2 — Original Data File [file 41419_2022_5062_MOESM2_ESM.ppt]

## Slide 1
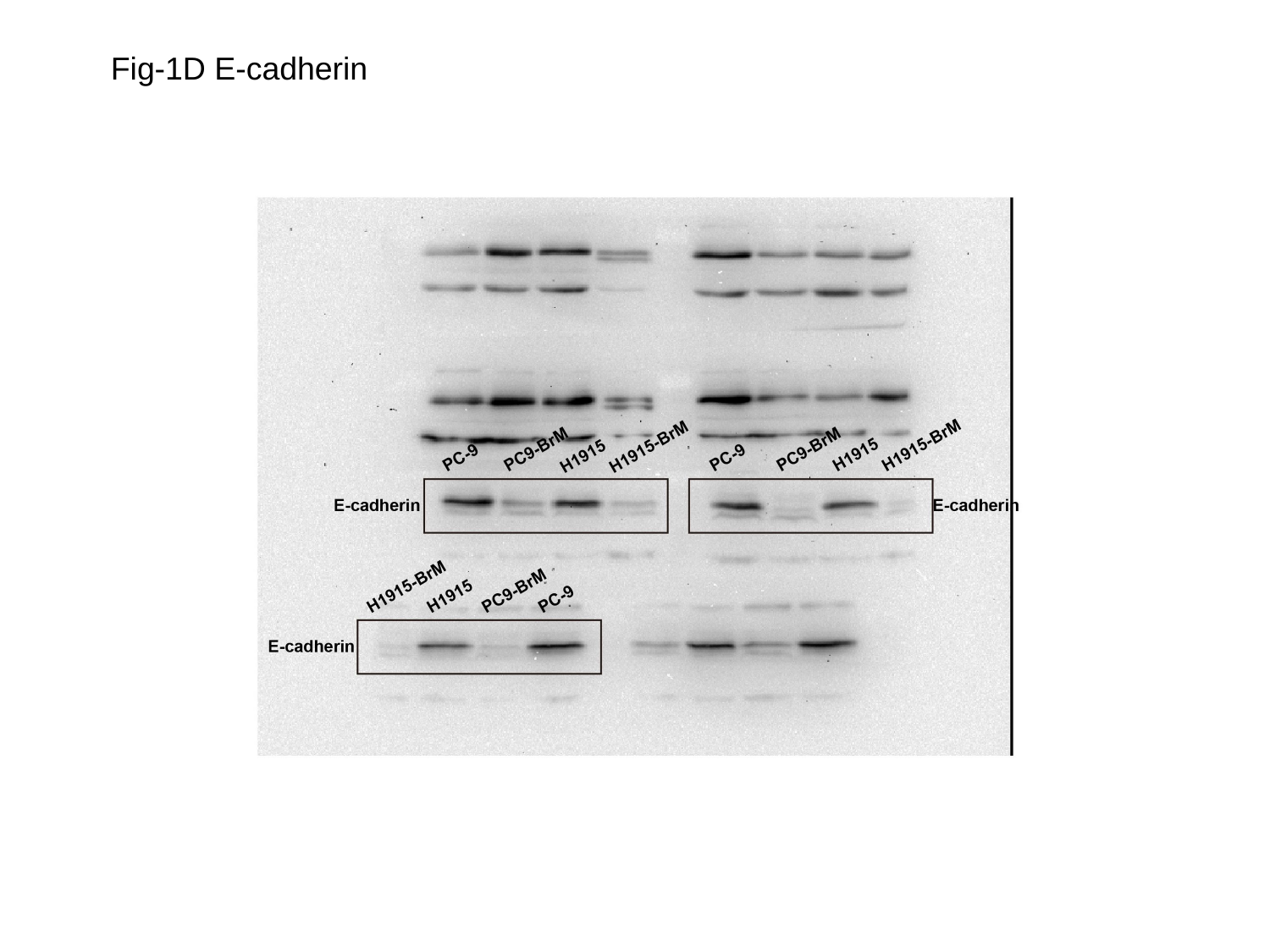

Fig-1D E-cadherin

## Slide 2
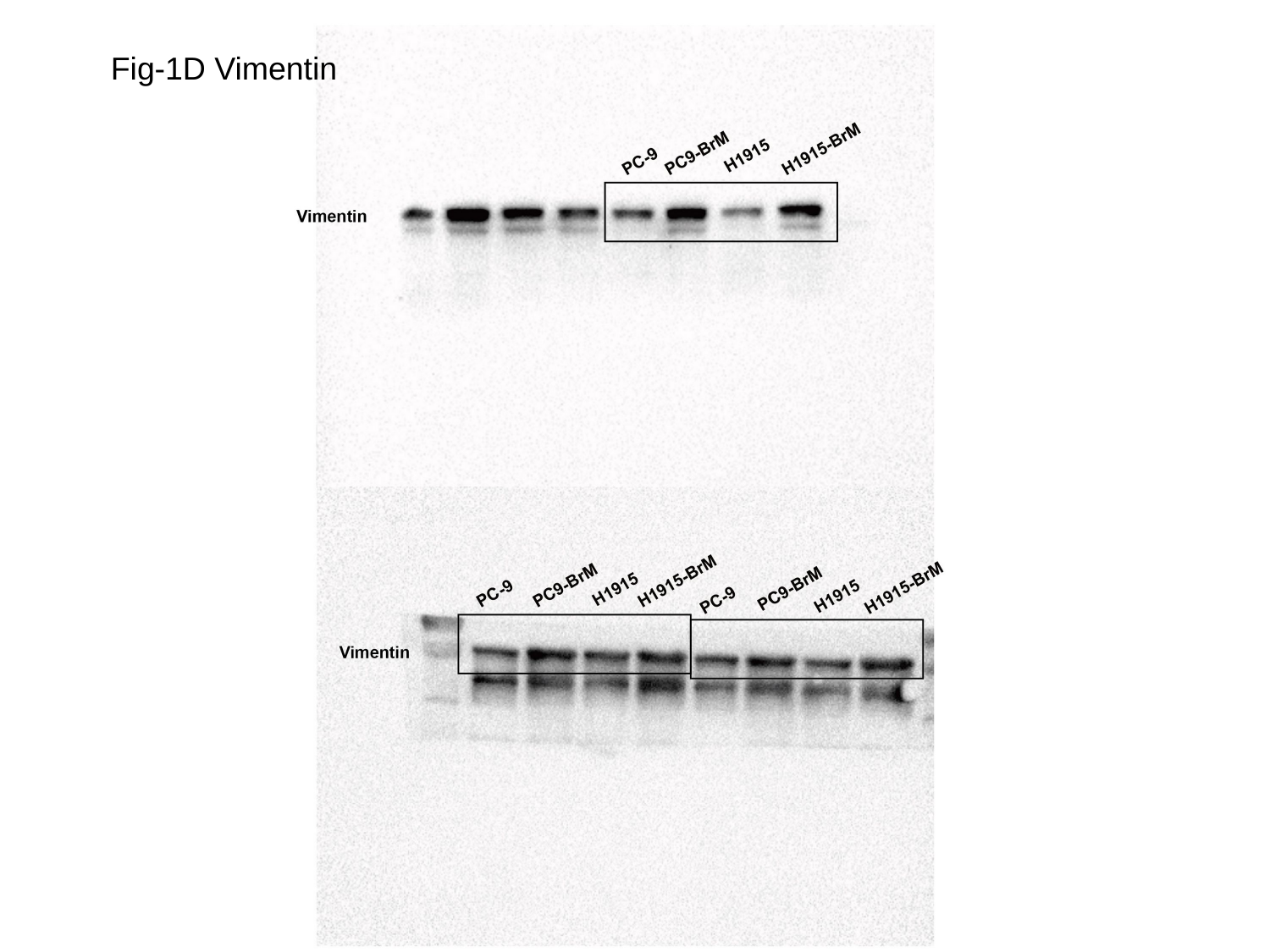

Fig-1D Vimentin

## Slide 3
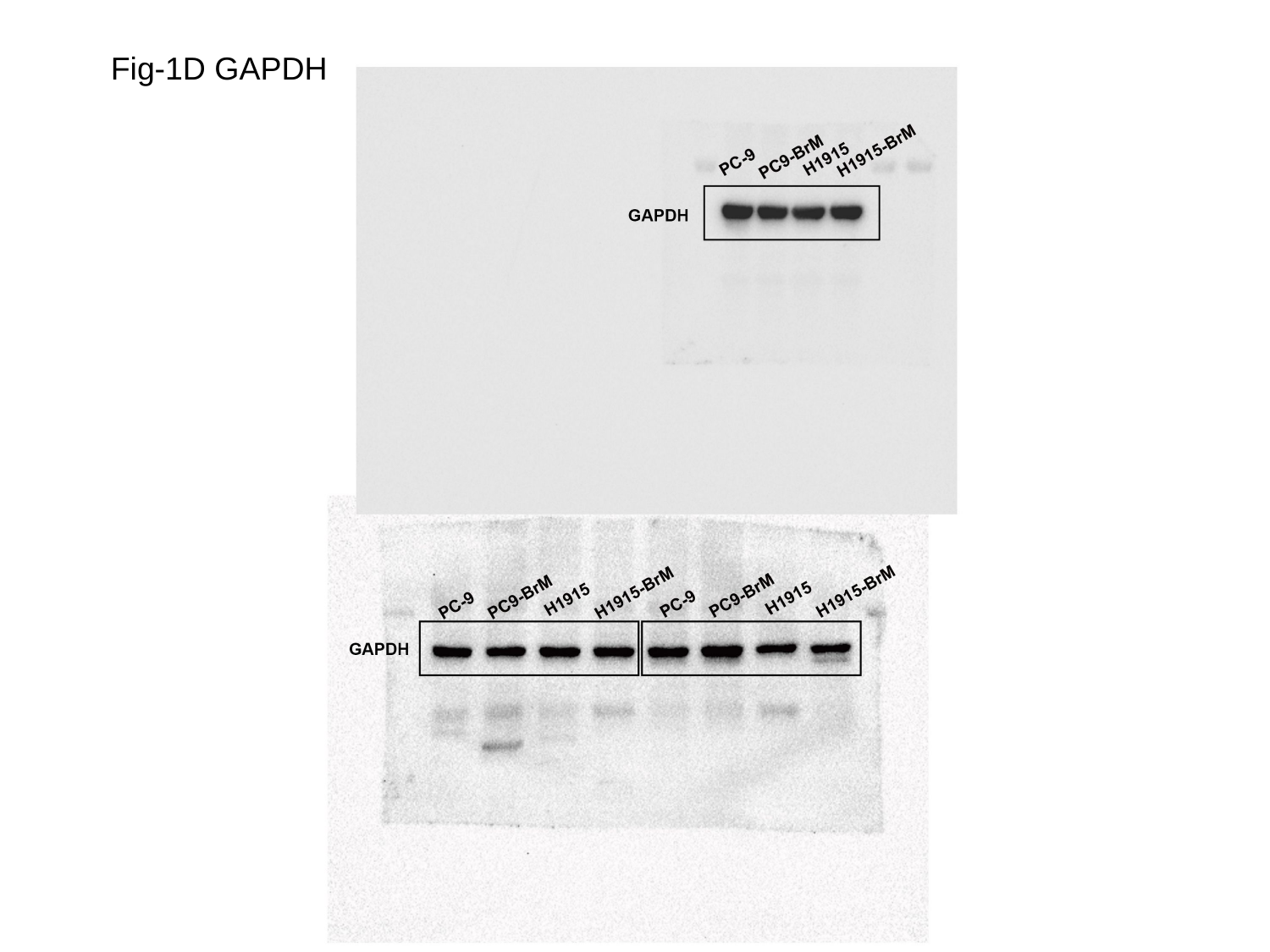

Fig-1D GAPDH

## Slide 4
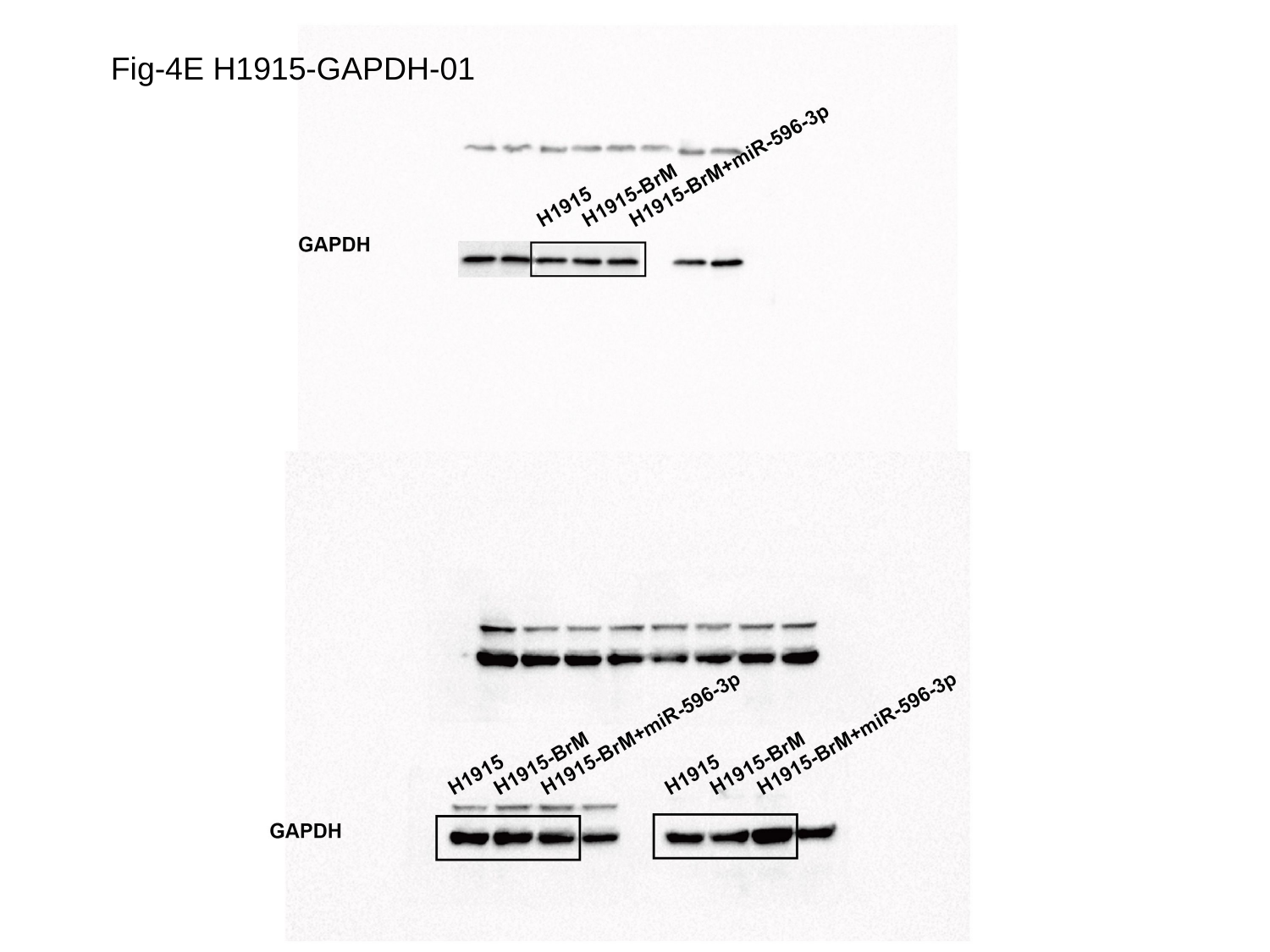

Fig-4E H1915-GAPDH-01

## Slide 5
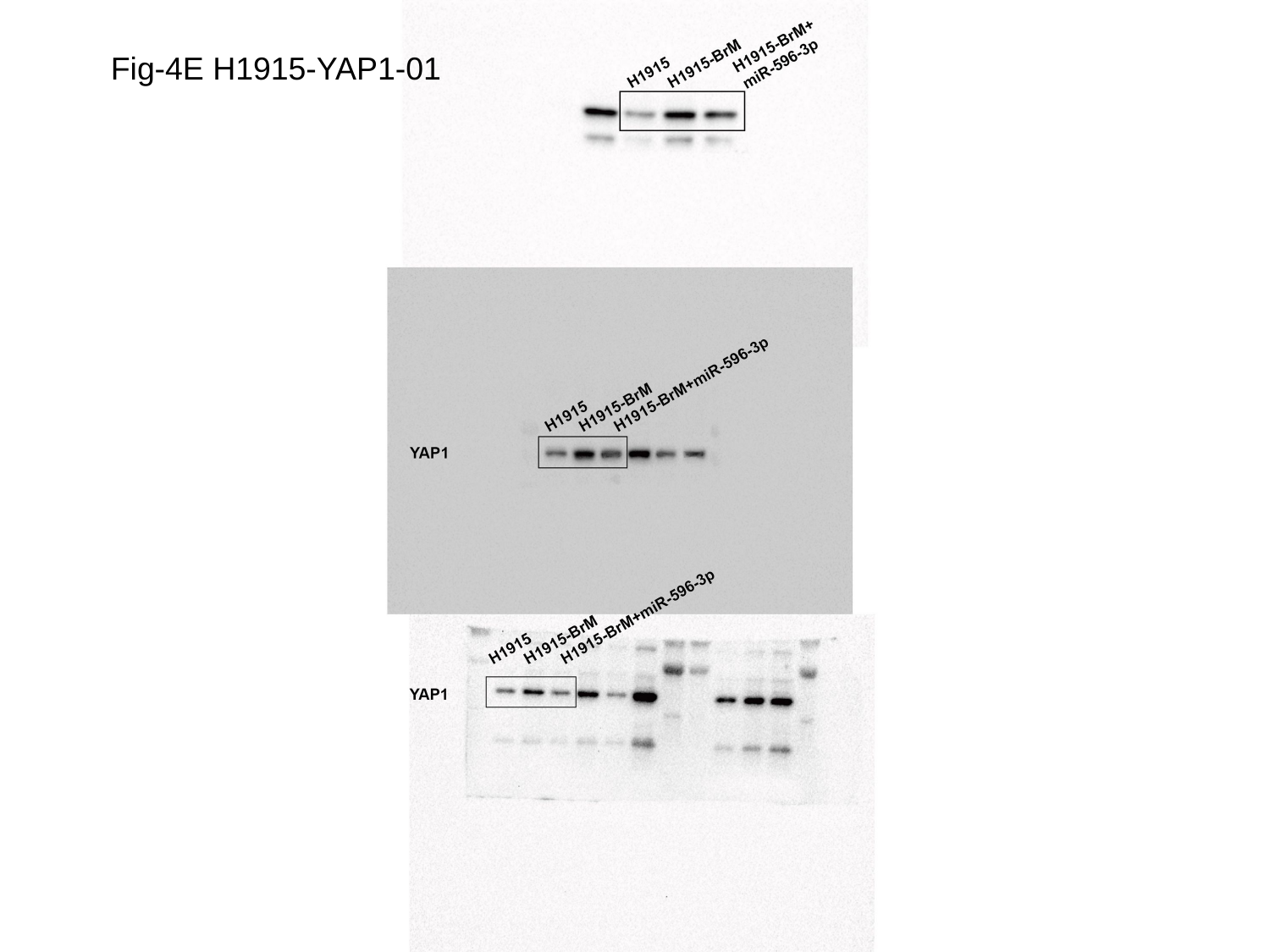

Fig-4E H1915-YAP1-01

## Slide 6
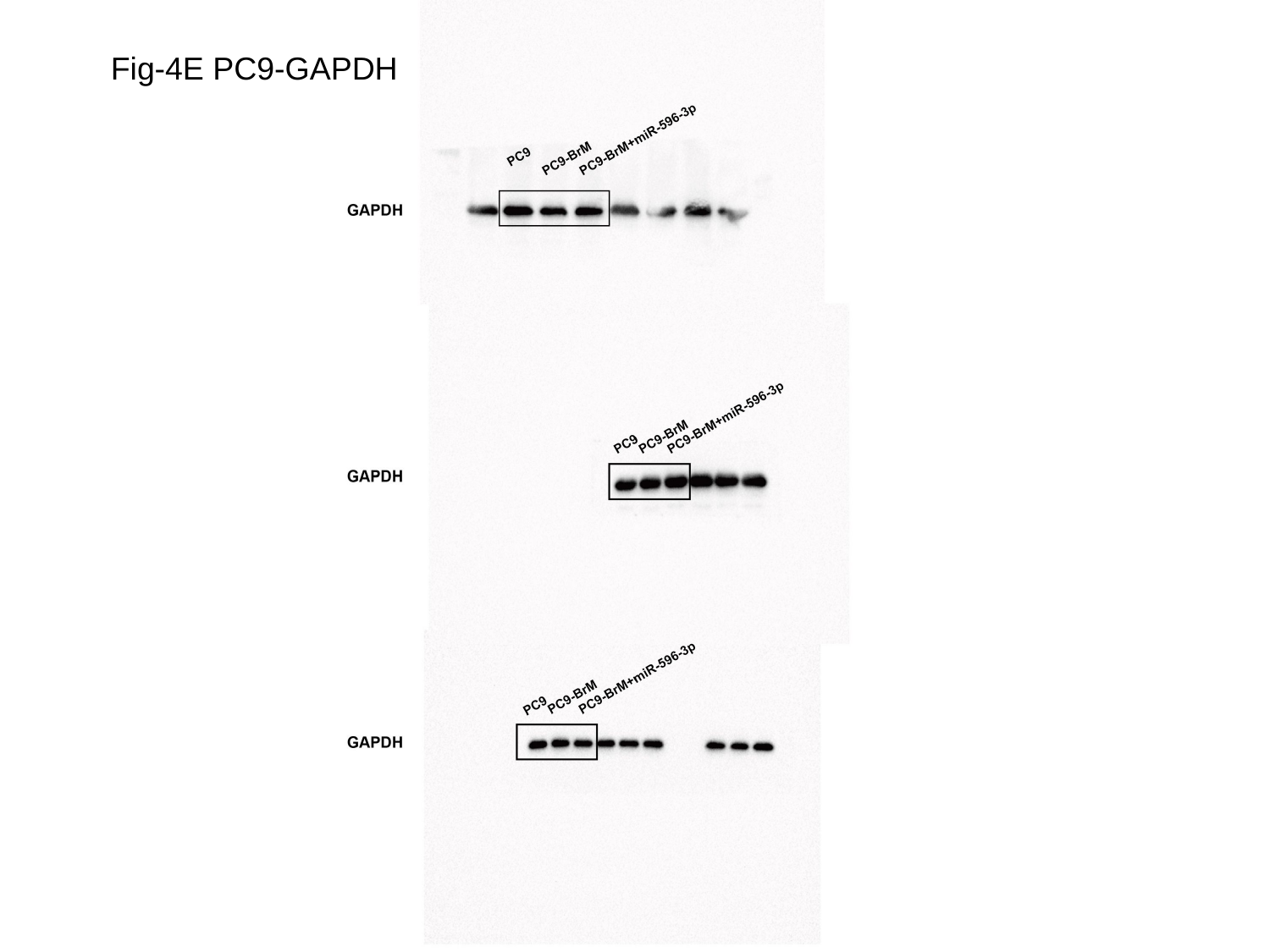

Fig-4E PC9-GAPDH

## Slide 7
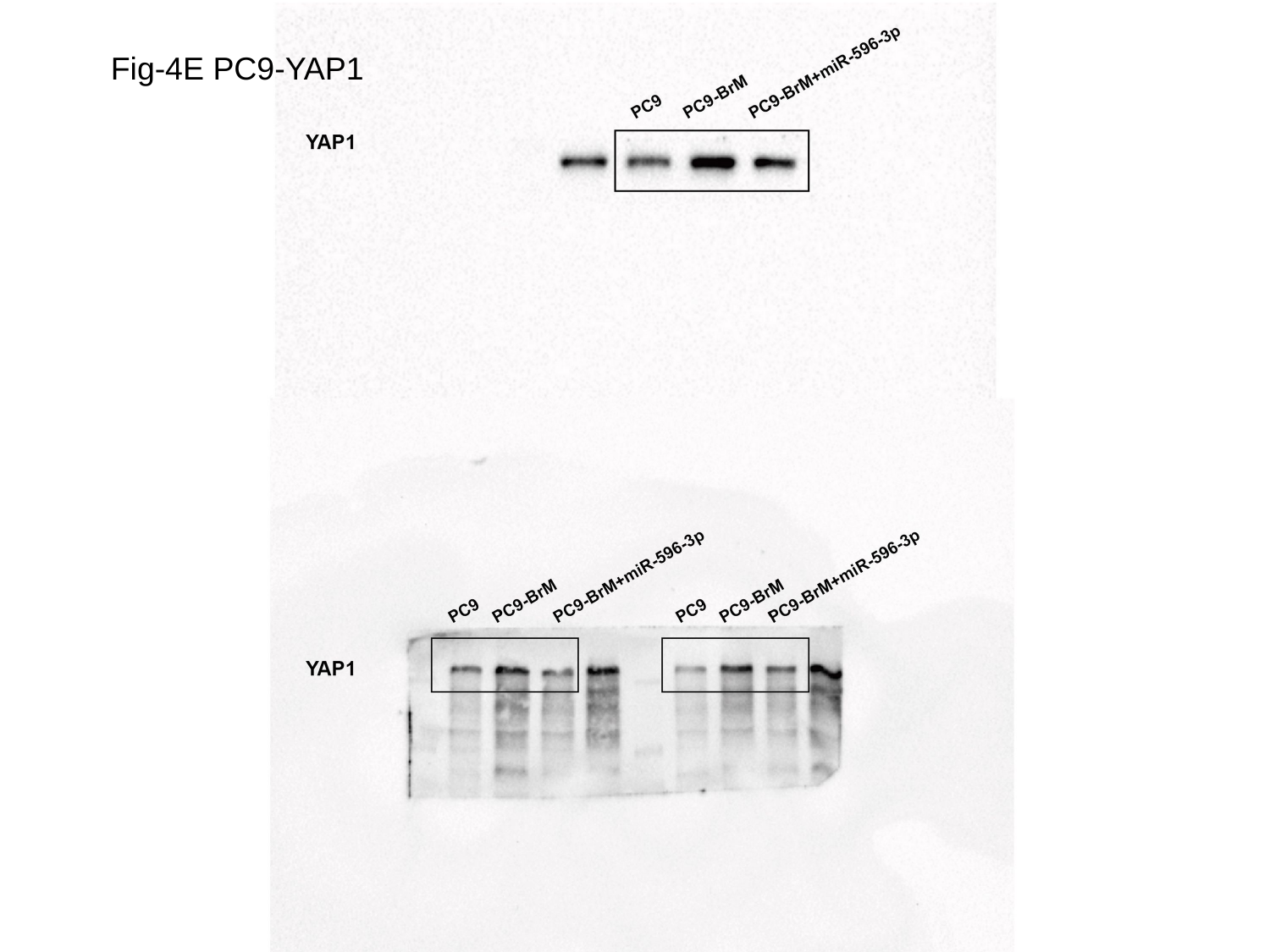

Fig-4E PC9-YAP1

## Slide 8
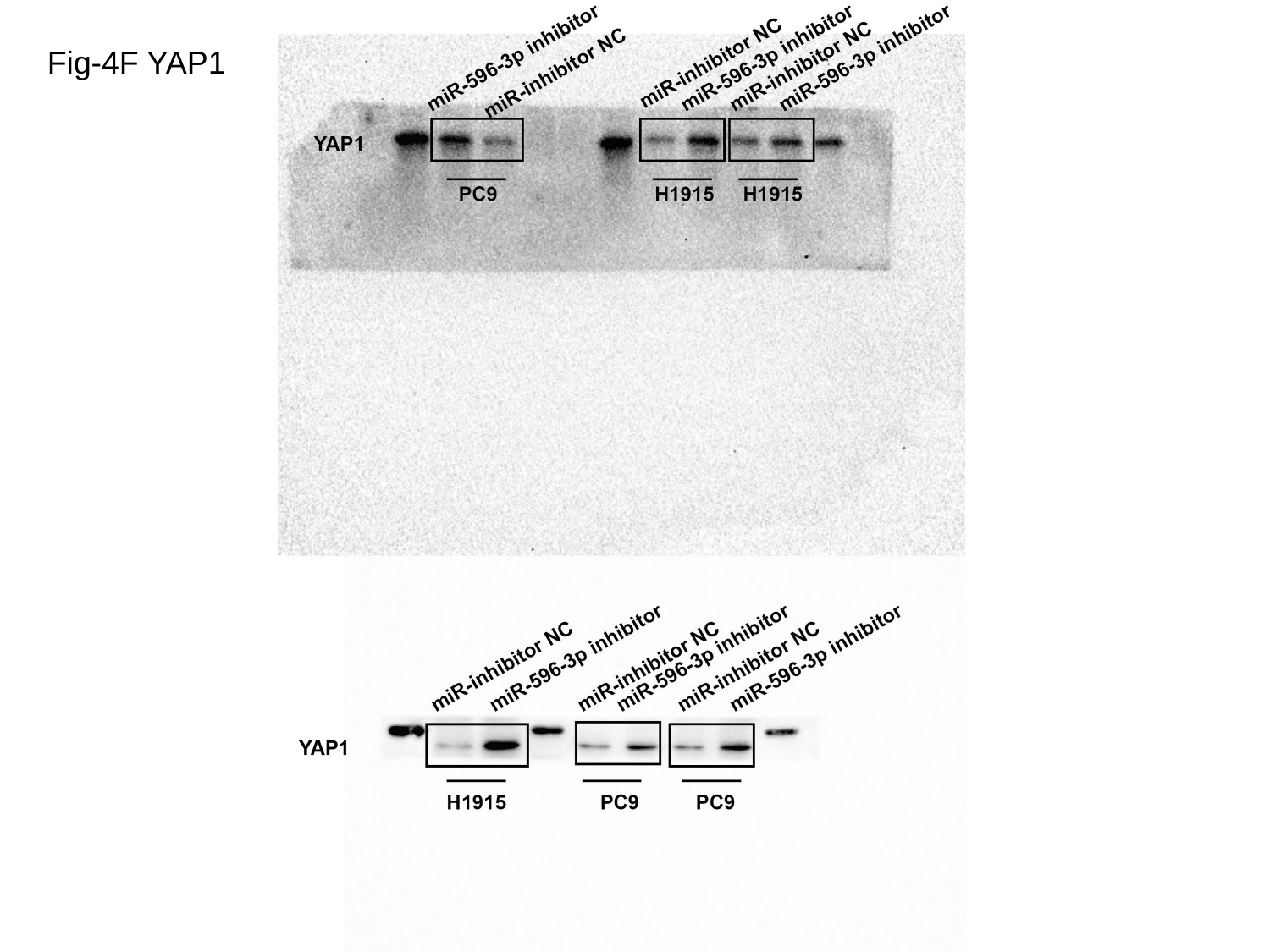

Fig-4F YAP1

## Slide 9
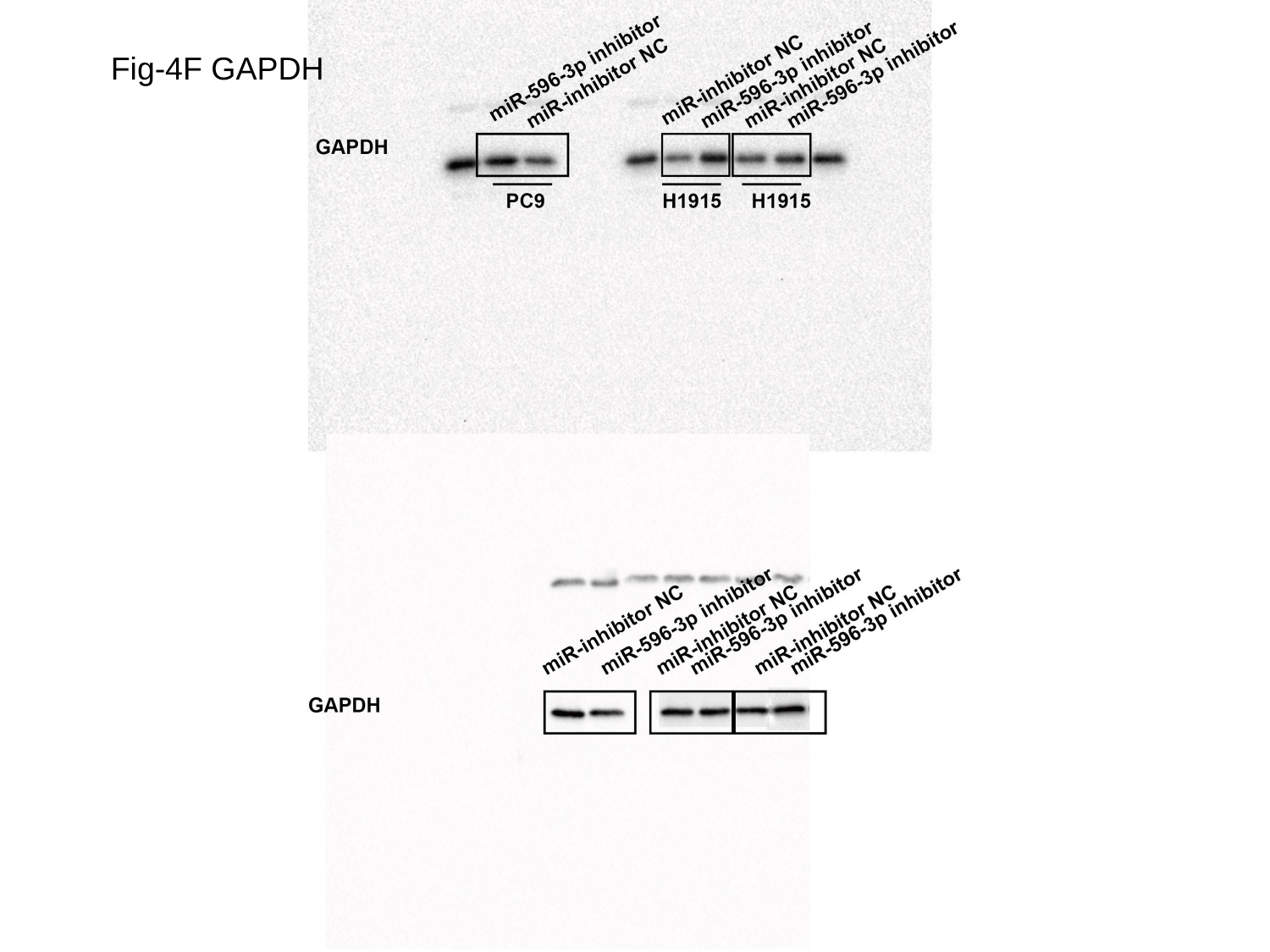

Fig-4F GAPDH

## Slide 10
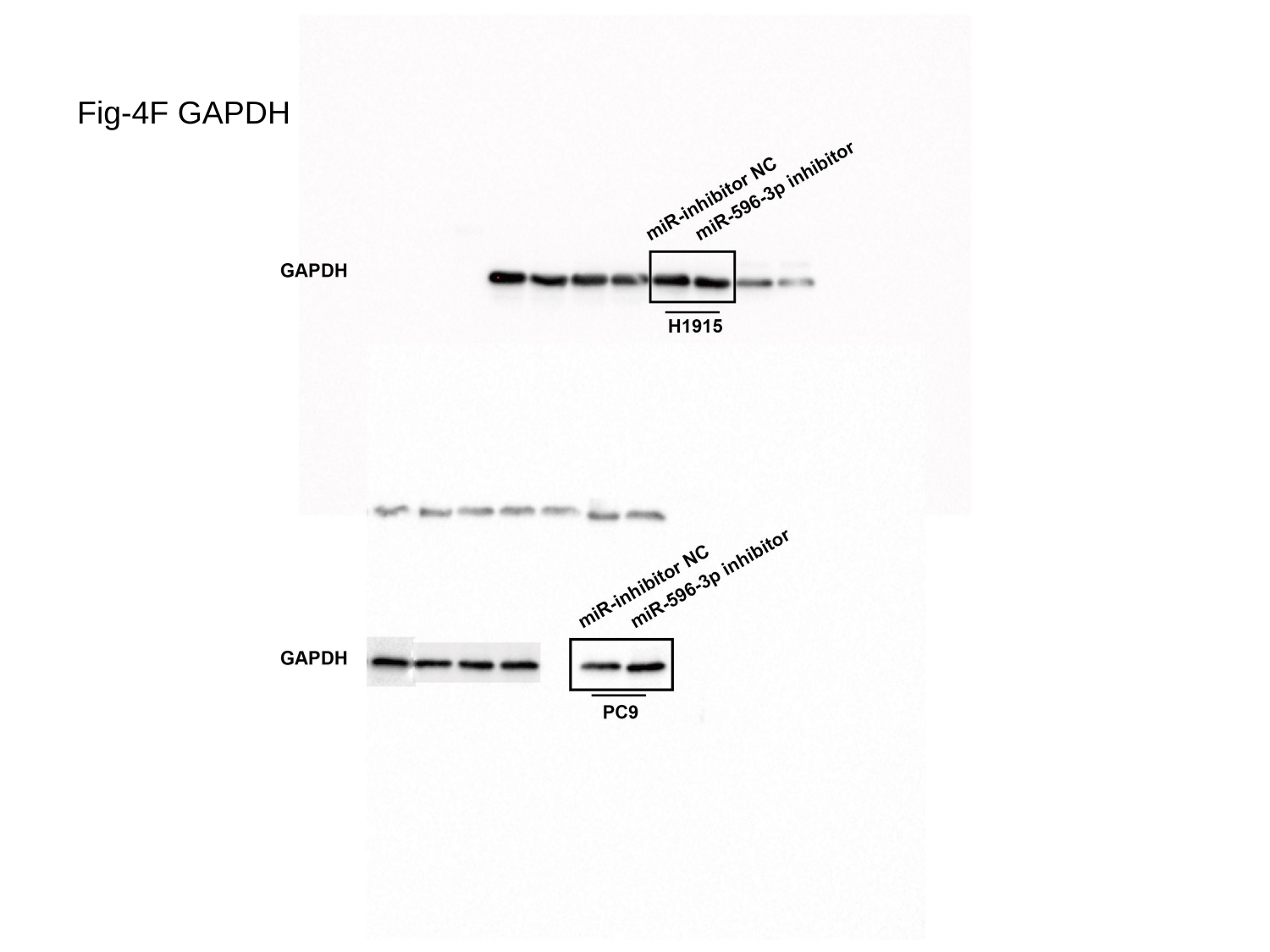

Fig-4F GAPDH

## Slide 11
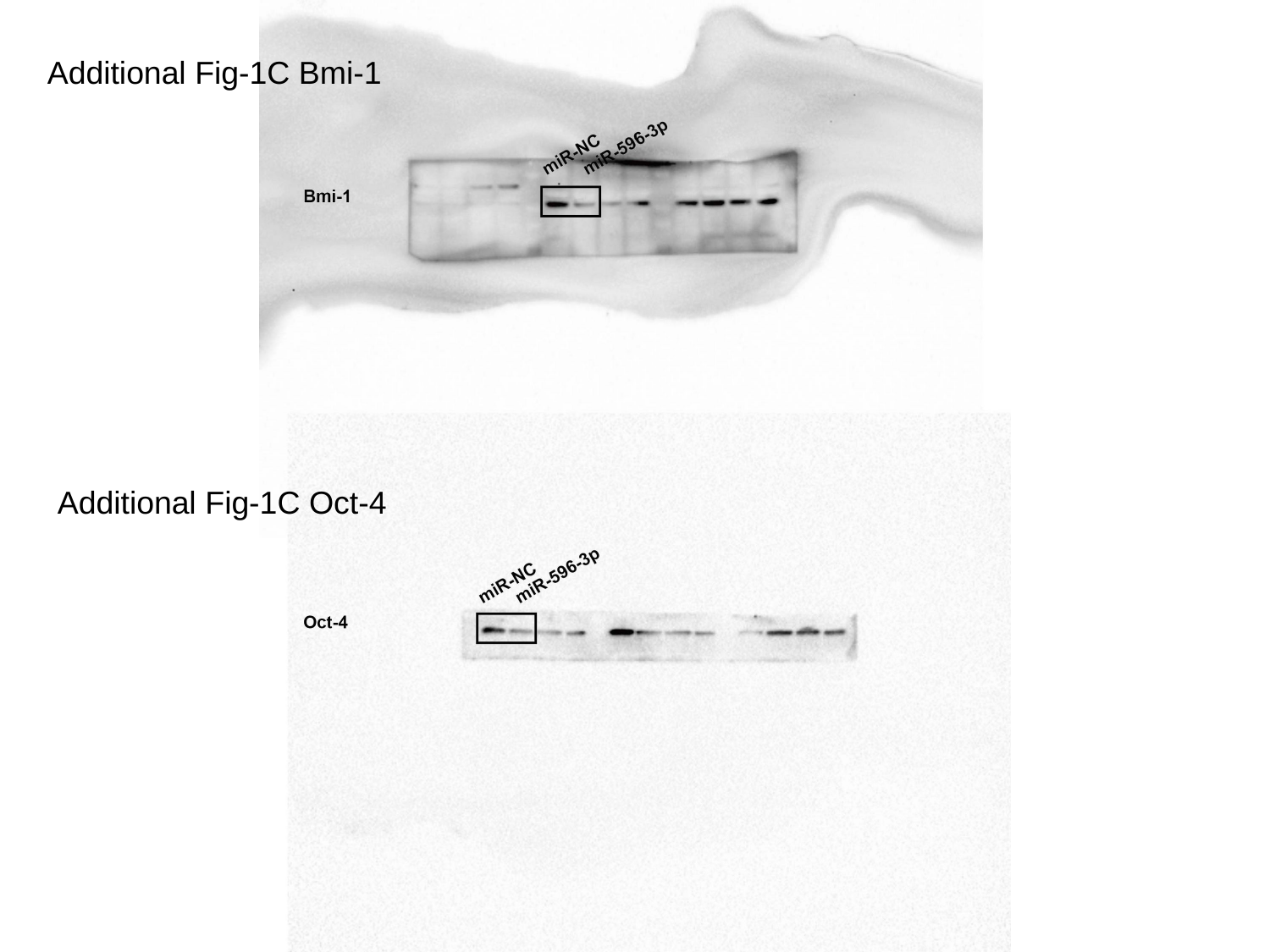

Additional Fig-1C Bmi-1
Additional Fig-1C Oct-4

## Slide 12
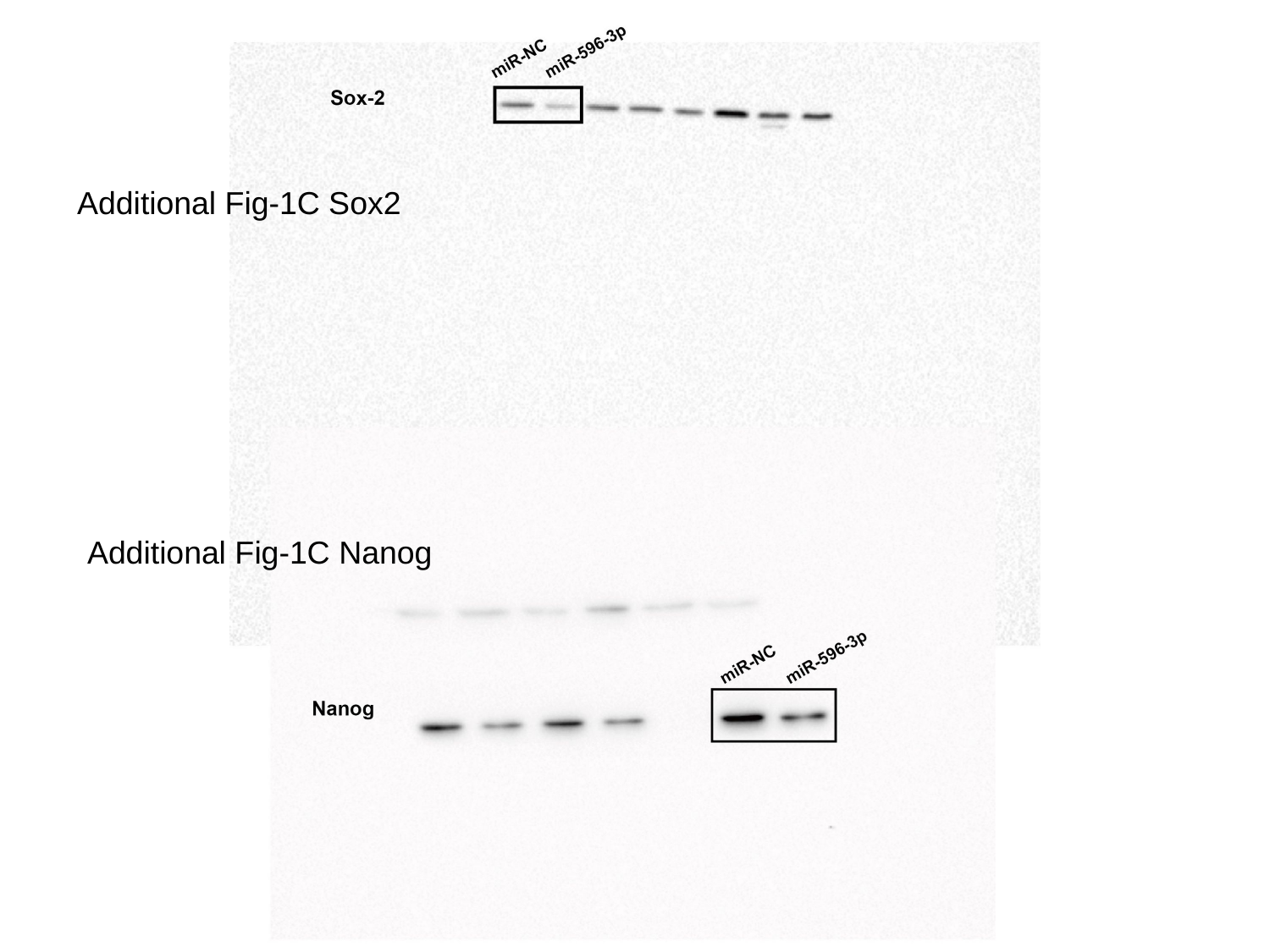

Additional Fig-1C Sox2
Additional Fig-1C Nanog

## Slide 13
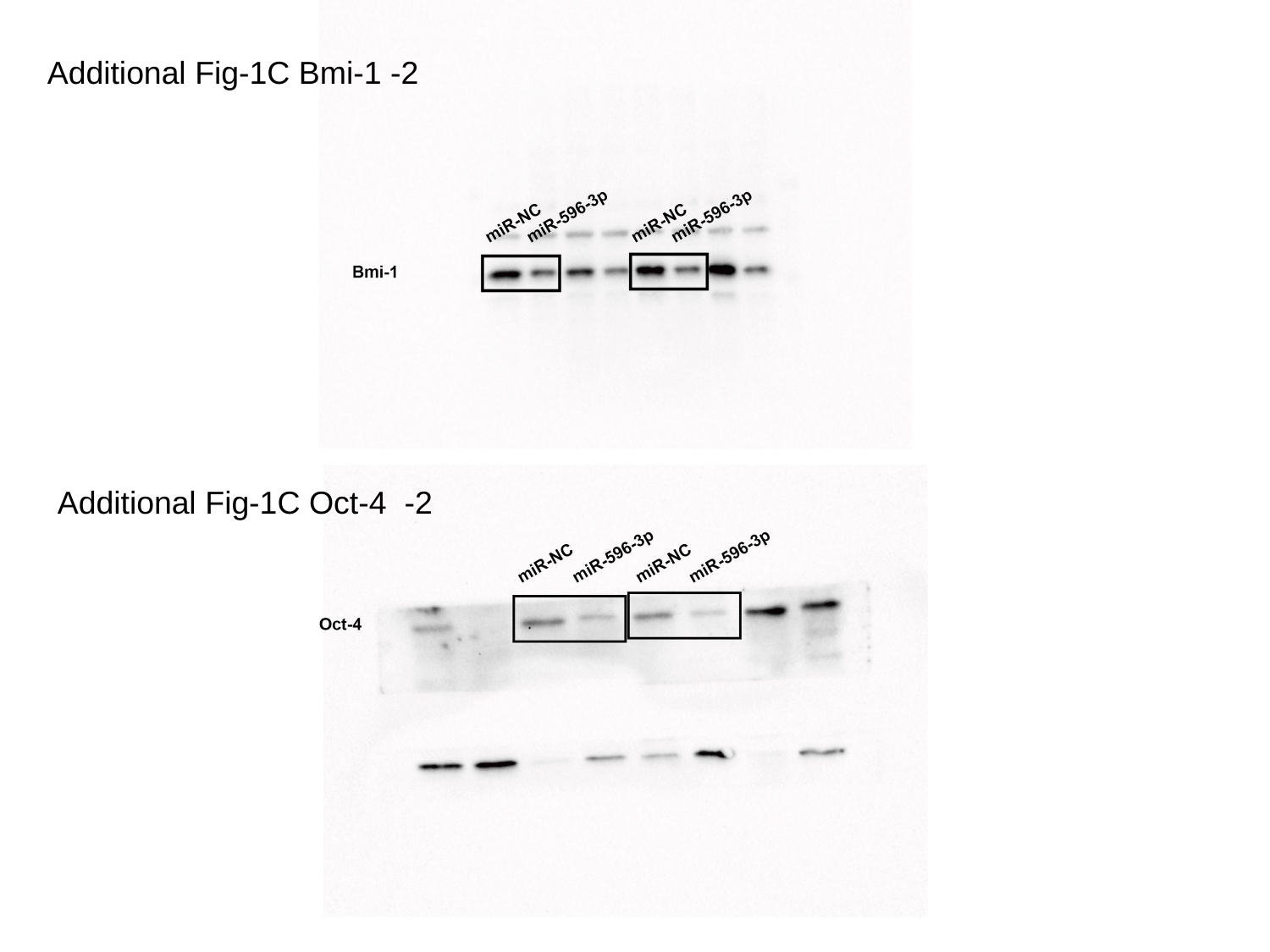

Additional Fig-1C Bmi-1 -2
Additional Fig-1C Oct-4 -2

## Slide 14
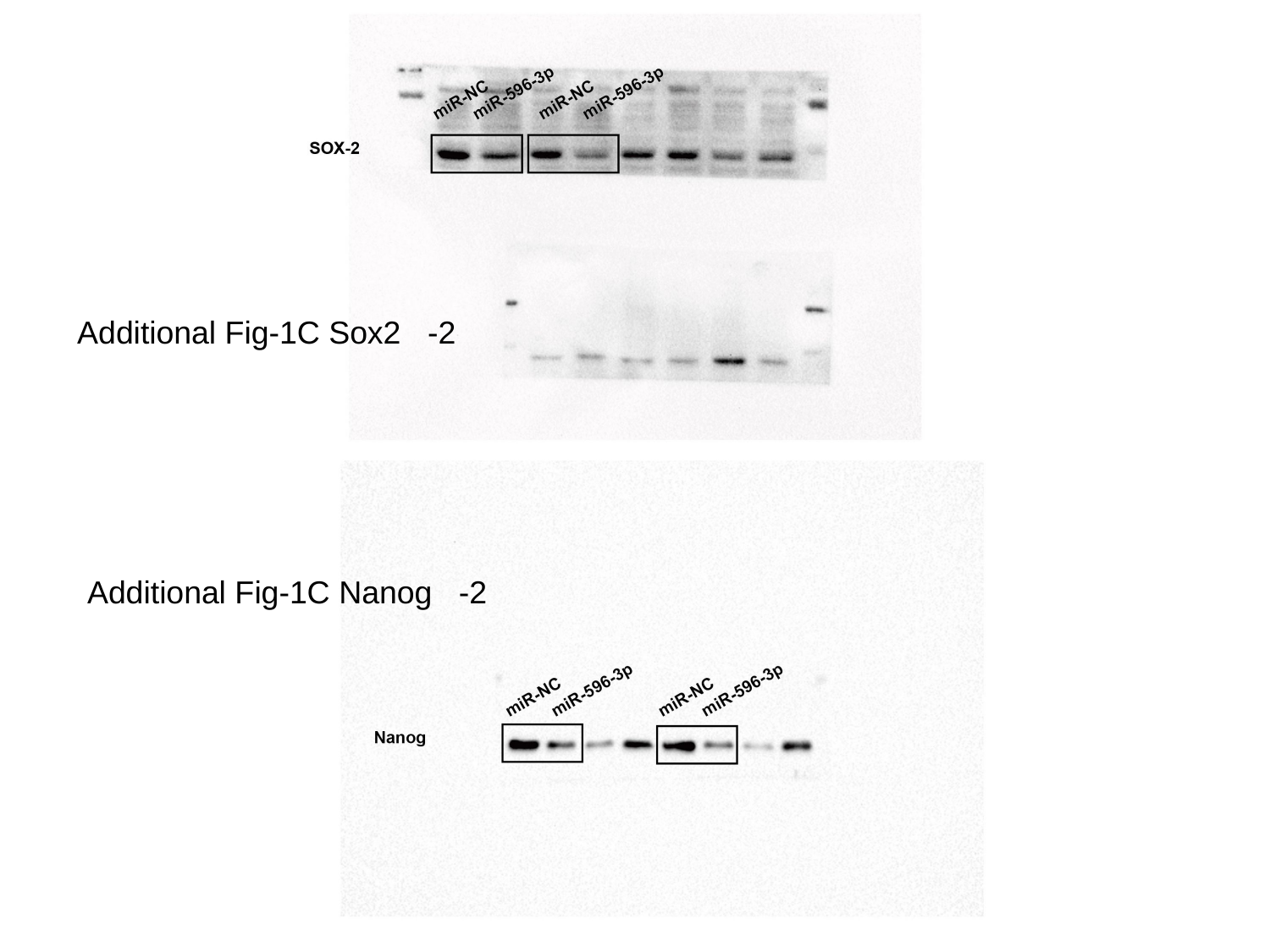

Additional Fig-1C Sox2 -2
Additional Fig-1C Nanog -2

## Slide 15
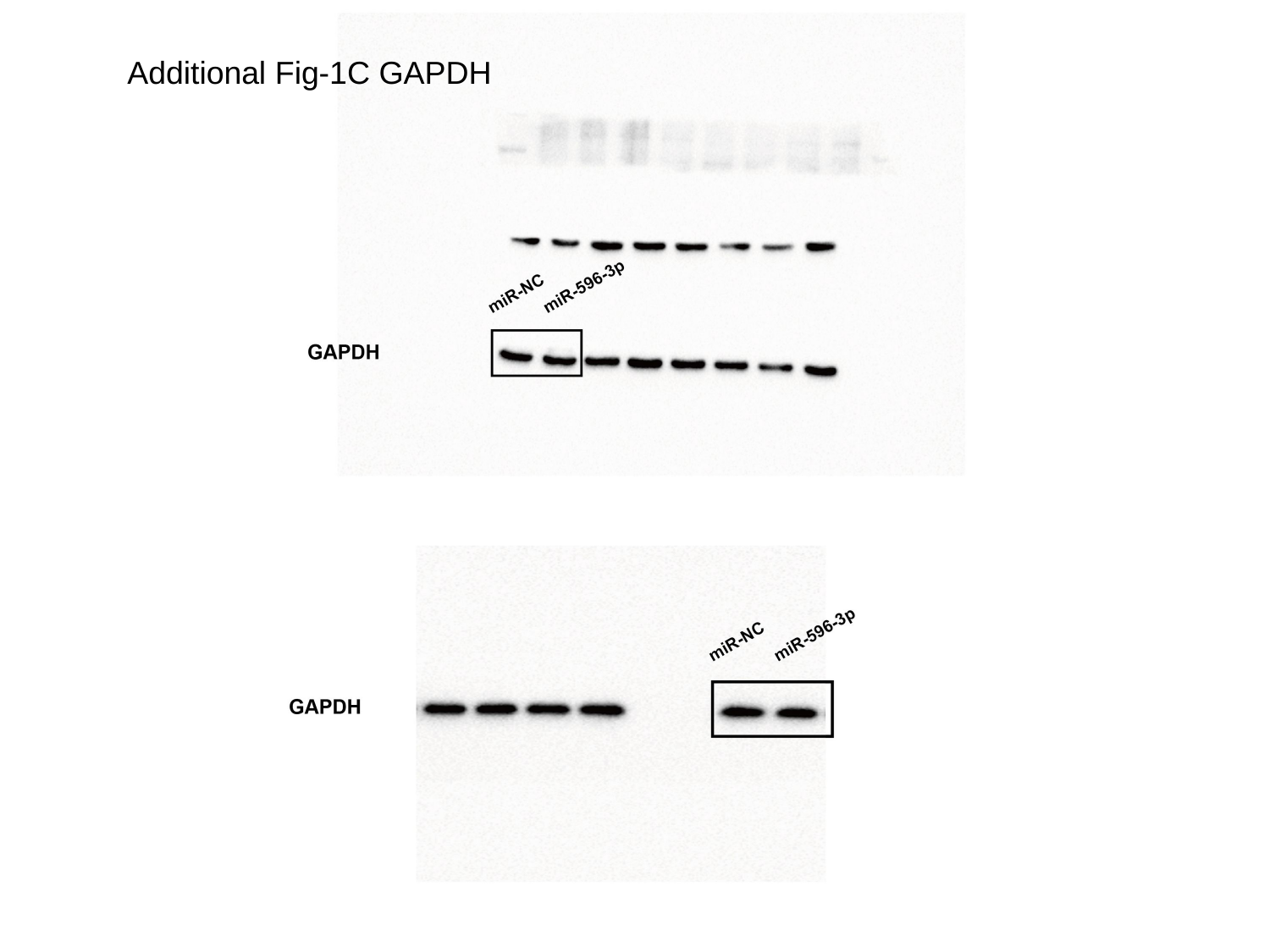

Additional Fig-1C GAPDH

## Slide 16
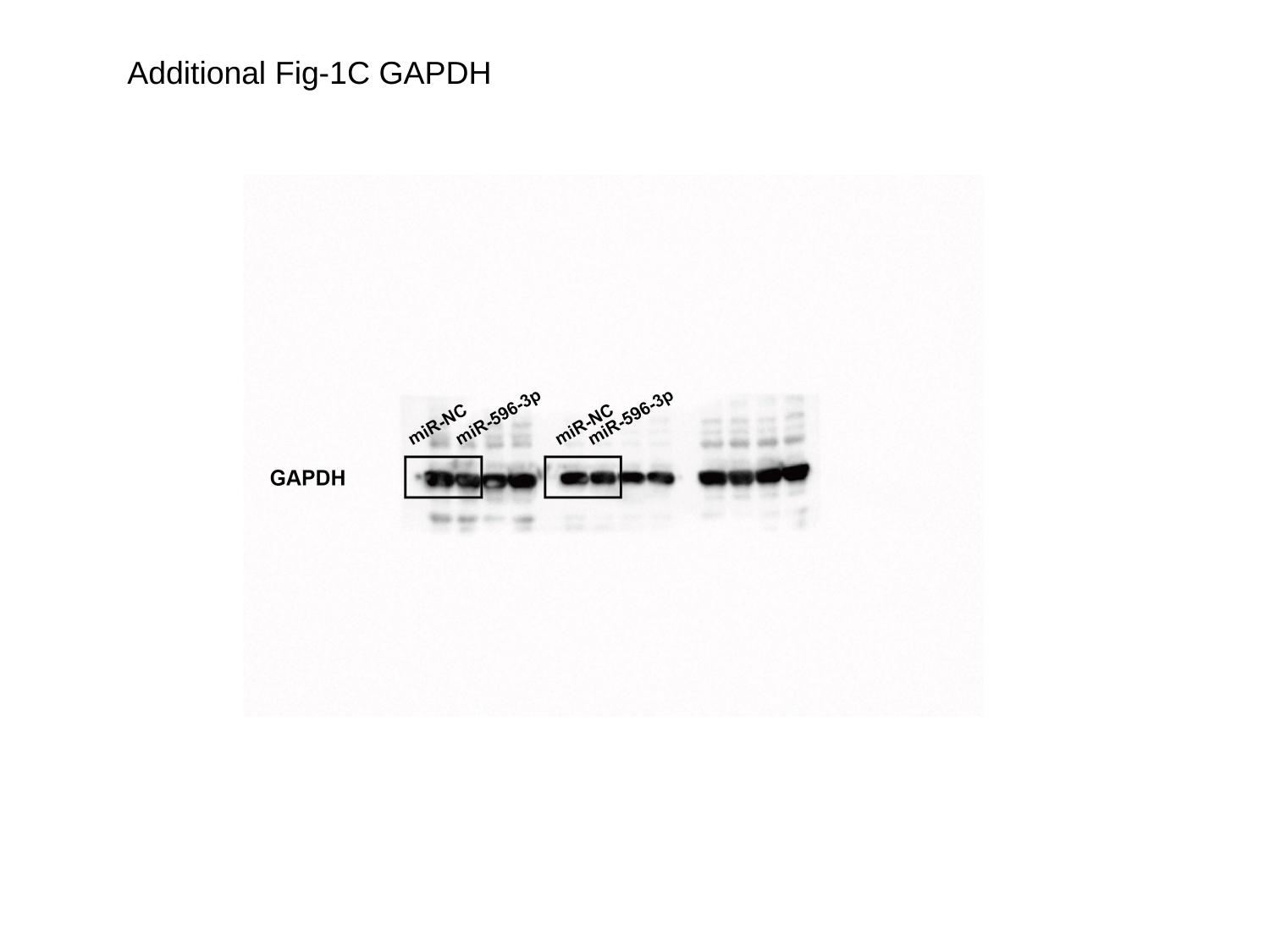

Additional Fig-1C GAPDH
