# Supplementary material for: miR-596-3p suppresses brain metastasis of non-small cell lung cancer by modulating YAP1 and IL-8
Source: Cell Death Dis. 2022 Aug 12;13(8):699. doi: 10.1038/s41419-022-05062-7 (PMC9374706; doi:10.1038/s41419-022-05062-7)
Supplement: Supplementary file 3 — Tracking chenge manuscript [file 41419_2022_5062_MOESM3_ESM.doc]

**miR-596-3p suppresses brain metastasis of non-small cell lung cancer by modulating YAP1 and IL-8**

Chenlong Li^1*^, Hongshan Zheng^1*^, Jinsheng Xiong^1*^, Yuxin Huang^5^, Haoyang Li^2,3,4^ , Hua Jin^1^, Siqi Ai^1^, Yingjie Wang^1^, Tianqi Su^1^, Guiyin Sun^1^, Xu Xiao^1^, Tianjiao Fu^1^, Yujie Wang^1^, Xin Gao^2#^, Peng Liang^1#^

^1^Department of Neurosurgery, Harbin Medical University Cancer Hospital, Harbin 150001, Heilongjiang, China;

^2^Computational Bioscience Research Center, King Abdullah University of Science and Technology (KAUST), Thuwal, 23955, Saudi Arabia;

^3^Cancer Systems Biology Center, the China-Japan Union Hospital, Jilin University, Changchun 130001, Jilin, China;

^4^College of Computer Science and Technology, Jilin University, Changchun 130001, Jilin, China;

^5^Heilongjiang Tuomeng Technology Co.Ltd., Harbin 150040, Heilongjiang, China.

**^*^**These authors contributed equally to this work.

***#Correspondence to:***

Professor. Peng Liang,

Department of Neurosurgery, Harbin Medical University Cancer hospital, No.150, Haping Road, Nangang District, Harbin 150001, Heilongjiang, China. TEL/Fax:+86-451-85718215. E-mail: [liangpengd@yahoo.com](mailto:liangpengd@yahoo.com)

Professor. Xin Gao,

Computational Bioscience Research Center, King Abdullah University of Science and Technology (KAUST), Thuwal, 23955, Saudi Arabia. E-mail: xin.gao@kaust.edu.sa

**Running Title: miR-596-3p suppresses brain metastasis of NSCLC**

**Keywords:** miR-596-3p; YAP1; NSCLC; brain metastasis

Abstract

Brain metastasis (BM) frequently occurs in advanced non-small cell lung cancer (NSCLC) and is associated with poor [clinical prognosis](../../../../F:/%25E8%25BD%25AF%25E4%25BB%25B6%25E5%25AE%2589%25E8%25A3%2585/Youdao/Dict/7.2.0.0703/resultui/dict/javascript:%3B). Due to the location of metastatic lesions, the surgical resection is limited and the chemotherapy is ineffective because of the existence of the blood brain barrier (BBB). Therefore, it is essential to enhance our understanding about the underlying mechanisms associated with brain metastasis in NSCLC. In the present study, we explored the RNA-Seq data of brain metastasis cells from the GEO database, and extracted RNA collected from primary NSCLC tumors as well as paired brain metastatic lesions followed by microRNA PCR array. Meanwhile, we improved the *in vivo* model and constructed a cancer stem cell-derived transplantation model of brain metastasis in mice. Our data indicated that the level of miR-596-3p is high in primary NSCLC tumors, but significantly downregulated in the brain metastatic lesion. The prediction target of microRNA suggested that miR-596-3p was considered to modulate two genes essential in the brain invasion process, YAP1 and IL-8 that restrain the invasion of cancer cells and permeability of BBB, respectively. Moreover, *in vivo* experiments suggested that our model mimics the clinical aspect of NSCLC and improves the success ratio of brain metastasis model. The results demonstrated that miR-596-3p significantly inhibited the capacity of NSCLC cells to metastasize to the brain. Furthermore, these finding elucidated that miR-596-3p exerts a critical role in brain metastasis of NSCLC by modulating the YAP1-IL8 network, and this miRNA axis may provide a potential therapeutic strategy for brain metastasis.

**Background**

Brain metastases (BM) are a severe clinical issue which tends to occur late in the progression of the primary cancer, and are associated with high morbidity and poor patient prognosis [1,2]. Lung cancer is the leading cause of cancer-related brain metastases [3]. Non-small cell lung cancer (NSCLC) accounts for 80% of patients with the primary lung cancer and approximately 30%-50% of patients developed brain metastases during the course of their progression [4]. Despite the improvements in the diagnosis and treatment, the median survival for brain metastases patients is about 2-3 and 4-6 months in untreated and treated patients, respectively [5,6]. Most chemotherapy drugs targeted primary tumors failed to inhibit the occurrence of brain metastatic tumors because of the existence of blood brain barrier (BBB) and the reduced chemosensitivity [7,8]. Both the ‘seed and soil hypothesis’ and ‘mechanical hypothesis’ have been proposed to the occurrence of brain metastases, but the real underlying mechanisms remain unclear [9,10].

Increasing evidence was spirited that non protein-coding RNAs are the [transcription](../../../../F:/%25E8%25BD%25AF%25E4%25BB%25B6%25E5%25AE%2589%25E8%25A3%2585/Youdao/Dict/7.3.0.0817/resultui/dict/%3Fkeyword=transcription)[product](../../../../F:/%25E8%25BD%25AF%25E4%25BB%25B6%25E5%25AE%2589%25E8%25A3%2585/Youdao/Dict/7.3.0.0817/resultui/dict/%3Fkeyword=product) for about 60-70% of human DNA [11]. It has been shown that small ncRNAs, microRNAs may act as a survival and prognosis [predictor](../../../../F:/%25E8%25BD%25AF%25E4%25BB%25B6%25E5%25AE%2589%25E8%25A3%2585/Youdao/Dict/7.3.0.0817/resultui/dict/javascript:%3B) in lung cancer [12]. Studies have suggested that the abnormal expression of miRNAs acts as vital roles in tumor occurrence and tumor development including metastasis [13], and it has been indicated that miR-4270, miR-423-3p, miR-330-3p, miR-423-5p and miR-197 mediate NSCLC brain metastasis [14-17]. Recent works have indicated the capacity to classify primary from secondary brain tumors and microRNA profiles were used to investigate the origin of metastatic lesions in brain [18,19]. It was found that the formation of brain metastasis lesion from NSCLC was regulated by the altered expression of genes involved in angiogenesis or extracellular matrix invasion [20]. However, tumor metastasis is a complex process and includes of the interactions of tumor cells with the microenvironment in the primary lung tumor tissues and metastatic lesions [9,20]. Although the critical role of microRNAs in NSCLC has been reported, exploring the underlying molecular mechanism of brain metastasis of NSCLC remains to be elucidated.

In the present study, we extracted RNA from tumor tissues collected from primary NSCLC tumors and paired brain metastatic lesions followed by microRNA profiling, which was closer to the real brain metastasis situation. In addition, our research suggested that the miR-596-3p could promote brain metastasis of NSCLC by inducing the expression of two essential genes in the brain metastasis process, YAP1 and IL-8, which together promote the increase in the penetration of tumor cells in the brain and act as a potential therapeutic target for brain metastasis intervention.

**Materials and methods**

**Tissue samples**

The formalin-fixed and paraffin-embedded (FFPE) samples from two groups of patients were studied, including 12 paired tissues (metastatic brain tumors and primary NSCLC samples from the same patient) and 12 primary NSCLC tissues without brain metastasis. All NSCLC patients with brain metastasis only observed the brain metastasis and no metastasis to other organs has been observed. The blood samples were collected from healthy donors (n=30) and the patients with early stage of NSCLC (n=30). All FFPE tissues were collected from Harbin Medical University Cancer Hospital between January 2013 to January 2019. All patients provided signed informed consent, and all procedures were performed in accordance with the principles outlined in The Declaration of Helsinki. The study was approved by the Institutional Review Board of Harbin Medical University.

**Generation of brain metastatic sublines PC9-BrM and H1915-BrM**

Brain is the most common site of metastasis from NSCLCs [21]. The NSCLC cell line PC-9 was purchased from Riken Cell Bank (Riken Cell Bank, Tsukuba, Japan) and H1915 was obtained from American Type Culture Collection (ATCC; Rockville, MD, USA), were cultured in RPMI-1640 medium (Hyclone, USA) with 10% Fetal bovine serum (FBS) and 100 U/mL penicillin/streptomycin (Sigma, st Louis, MO, USA). We obtained A549, H460 NSCLC cell lines, human brain microvascular endothelial cell (HBMEC) and normal human astrocyte (NHA) from ATCC. All cells were seeded in RPMI-1640 medium (HyClone; GE Healthcare Life Sciences, Logan, UT, USA) supplemented with 10% fetal-bovine serum (Gibco; Thermo Fisher Scientific, Inc., Los Angeles CA, USA) in a 37ºC, 5% CO_2_ humidified condition.

In this research, we conducted the *in vivo* selection experiments to extract metastatic cells from the parental PC9 and H1915 human NSCLC cell lines. The cells were widely used to establish the brain metastasis subcell line. We initially inoculated the mice with PC9 and H1915 cells via the left cardiac ventricle. Tumour development was monitored by weekly bioluminescence imaging using the Bruker In-Vivo FX PRO Imaging System (Bruker Corporation, Germany). Brain metastatic lesions in mice were confirmed by histological analysis after the mice were sacrificed using CO_2_ (28% volume displacement per min) and the brain were removed sterile conditions [22]. The mice brain was minced and cultured in RPMI-1640 medium adding with antibiotic agents and 10% fetal bovine serum (FBS, Gibco; Thermo Fisher Scientific, Inc., Los Angeles CA, USA). The cells were briefly centrifuged and seeded in a culture medium supplement with 50 μg/mL Zeocin (Gibco, Thermo Fisher Scientific, Inc., Los Angeles CA, USA) to confluence on a 10-cm dish. We repeated this injection-isolation-expansion process for three additional times and acquired the subcell line using this process as PC9-BrM and H1915-BrM.

**microRNA microarray profiling**

The primary NSCLC samples and brain metastatic lesions of NSCLC patients were
collected from FFPE samples and prepared for 10 µm thickness. Total RNAs were isolated from FFPE samples by employing the miRNeasy FFPE kit (cat no.217504, Qiagen Group, China). Expression profiling of microRNA was performed by using the miScript miRNA PCR Array (cat no.331231, Qiagen Group, China). The visualization data were displayed using HEMI 1.0 software.

**Data acquisition**

The RNA-seq data were collected from the Gene Expression Omnibus (GEO) database (series: GSE83132; GSE11969, https://www.ncbi.nlm.nih.gov/geo/query/acc.cgi?acc). The DEGseq algorithm was subjected to screen the differentially expressed genes with a fold change (FC)> 2, false discovery rate (FDR)< 0.05. Gene Ontology (GO) analysis was conducted according to the GO annotations from the Database for Annotation, Visualization and Integrated Discovery (<http://david.abcc.ncifcrf.gov/>) online tool. Gene Set Enrichment Analysis (GSEA) was used to characterize biological functions associated with miR-596-3p expression sets from Broad Institute Molecular Signature Database (MSigDB).

**Reverse Transcription Quantitative PCR (RT-qPCR)**

Total RNA was extracted from tissues/FFPE samples and cultured cells using TRizol reagent/the miRNeasy FFPE kit (cat no.217504, Qiagen Group, China) according to the manufacturer’s instruction. RT-qPCR was conducted using SYBR®Premix Ex Taq™Ⅱ(cat no. RR820B; Takara, Dalian, China) in the ABI PRISM® 7300 real-time PCR system (Applied Biosystems, Foster City, CA, USA). U6 was used as an internal reference of miRNA. GAPDH was used as an endogenous control of YAP1. The primer sequences were listed in Table 1. The relative expression levels were analyzed using the 2^-ΔΔCt^ method [23].

**Plasmid transfection.**

The tumor cell lines were maintained in RPMI-1640 medium supplemented with 10% FBS at 37˚C in a 5% CO_2_ condition. siRNAs, pcDNA3.1 and miRNA mimics (50 μM) were purchased from Shanghai GeneChem Co., Ltd.. Cells were transfected with using Lipofectamine® 2000 (Invitrogen; Thermo Fisher Scientific, Inc., USA; cat. no. 11668030) when the cell confluence was about 70-80%, and cultured at 37˚C for another 4h. The supernatant medium was removed and added with fresh complete DMEM medium, and subsequent experiments were conducted at 24h post-transfection.

**Dual-luciferase reporter assay**

The luciferase reporter assay was employed to evaluate the potential binding between miR‑200a‑3p and YAP1. The psiCHECK2 vector (GeneChem, Inc., Shanghai, China) was used to construct a YAP1 3'‑untranslated region (UTR)‑containing reporter. The 3'‑UTR of YAP1, as well as its wild‑type (WT) and mutant (MUT) binding sites with miR-596-3p, were amplified and cloned into luciferase reporter vectors.Human 293T cells were cultured in 6-well plates and the miR-596-3p mimics/miR-NC (20 μM) were co-transfected with psiCHECK2-YAP1/IL-8 wild type (WT) or mutant (MUT) by Lipofectamine**®**2000 (Invitrogen; Thermo Fisher Scientific, Inc., USA; cat. no. 11668030). The supernatant medium was removed at 4h post-transfection. After 48h, the activity of Firefly luciferase was detected using a Dual-Luciferase Reporter Assay System (cat. no. RG027; Beyotime Institute of Biotechnology, Shanghai, China) according to the manufacturers’ protocol. *Renilla* luciferase was employed as an internal control.

**Cell proliferation assay**

The proliferation of NSCLC cells was performed by using Cell Counting Kit-8 (Beyotime Institute of Biotechnology, Shanghai, China; CCK-8; cat. no. C0037). PC9-BrM cells were cultured in 96-well plates (2×10^3^ cells/well) at 37˚C under 5% CO_2_ atmosphere for 24 and 48. Then 10 µL of CCK-8 solution was fixed to the well and incubated for another 4h. The OD value was measured at 450 nm by the Tecan microplate reader (Infinite F50; Tecan Group, Ltd., Swiss).

**Transmigration assays and Matrigel invasion assay**For transmigration assay, the HBMEC cell (1×10^5^) and NHA cell (1×10^5^) were cultured on the upper (HBMEC) and bottom (NHA) membrane of a transwell chamber (Costar; Corning, Inc; pore size: 3 µm; cat. no. 3495) and grown to (>95%) confluency. Brain metastasis cells (5×10^5^) were labeled with Cell Tracker Green (Life Technologies, Carlsbad, CA, USA; cat.no. C7025) and were added with serum-free medium in the top inserts. The bottom chamber was fixed with medium containing 20% serum. After 24h, the labeled cells invaded via the membrane covered with the HBMEC cells were calculated under a fluorescent microscope (Nikon Corporation) at ×100 magnification.

For the matrigel-invasion assay, brain metastasis cells (5×10^5^) were fixed into Matrigel-precoated transwell chamber (Costar; Corning, Inc., USA; cat. no.3495) containing with serum-free medium. The bottom side was added with medium supplemented with 20% serum. The non-invading cells were removed by using a cotton swab. Then cells in the bottom chamber were fixed with 4% paraformaldehyde at room temperature for 15 min and stained with Hematoxylin-Eosin/HE Staining Kit (Beijing Solarbio Science & Technology Co., Ltd.; cat. no. G1120) at room temperature. The invaded cells were photographed and calculated in five independent fields for each well under a light microscope (Nikon Corporation) at ×100 magnification.

**Western blotting**

#### The transfected cells were washed twice with PBS and collected by adding Thermo scientific RIPA buffer (Pierce, USA; cat.no.89900) with protease inhibitors. The PIERCE BCA protein assay kit was used to measure the protein concentration. The PVDF (Millipore, Billerica, MA, USA) membranes were blocked with 5% BSA at room temperature for 1h and incubated separately with relative primary antibody. Then they were incubated with horseradish peroxidase-conjugated goat anti-rabbit secondary antibody (1:1 000; Proteintech Group, Inc., Wuhan, China; cat.no. 10494-1-AP) for 1h. The relative protein expression levels on the PVDF membrane were detected with the ECL system with ChemiDocTM MP Imaging System and analyzed by Image Lab software V3.0 (Bio-Rad Laboratories, Inc.).

**Immunofluorescence analysis**

##### Cells slides were fixed with cold ethanol for 20 min and incubated with 0.1% Triton x-100 for 5 min at room temperature. The slides were washed with PBS and blocked with 5% BSA in PBS. Then cells were added with rabbit anti-human IL-8 (Table 2) and incubated for 1h at 4℃. After that samples were incubated with Rhodamine (TRITC)-conjugated Goat Anti-Rabbit IgG second antibody (1:1 000, ProteinTech Group, Inc., China; cat.no. SA00007-2). The nuclei were stained with 4’-6-diamidino-2-phenylindole (DAPI) for 15 min. Fluorescence images were captured by a fluorescent microscope (Nikon Corporation).

**Transendothelial electrical resistance (TEER) assay**BBB function of the *in vitro* BBB model was explored by the TEER assay. The resistance values (Ω) was measured in confluent HBMEC monolayers by employing an EVOM™ Epithelial Voltammeter (World Precision Instruments, Sarasota, FL, USA). The HBMEC cells (5×10^5^) and NHA cells (5×10^5^) were placed in both membrane of the upper insert and grown to confluency. Conditioned medium (CM) from PC9-BrM and PC9-BrM+miR-596-3p were seeded into the upper insert and TEER was detected every 12 h.

**ELISA assay**

ELISA assay was performed to measure the level of MMP-2 in PC9-BrM cells by human MMP-2 pre-coated ELISA kit (Beijing Solarbio Science & Technology Co., Ltd., China; cat.no.[SEKH-0253](http://www.solarbio.com/goods-38600.html); ) in accordance with the manufacture’s instruction. Data was measured using Tecan microplate reader (Infinite F50; Tecan Group, Ltd., USA).

**Cytokine array**

#### Cytokine array in PC9-BrM transfected with miR-596-3p conditioned medium (CM) was conducted using Cytokine Antibody Array I kit from Abcam (Abcam, Cambridge, United Kingdom; cat.no. Ab133998). After blocking the array membranes for 30 min, the membranes were incubated with 1 mL of CM at room temperature for 1.5h. Then the membrane was washed with buffer, and fixed with primary biotin-conjugated antibody to the membrane and incubated for another 2h. Then the horseradish peroxidase-conjugated streptavidin was fixed with the membrane, we added the detection buffer on the membrane, employing Image Lab software V3.0 (Bio-Rad Laboratories, Inc., USA). The data was measured by the Image J software.

**Sphere formation assay**

Brain metastatic PC9-BrM cells were cultured in DMEM medium supplemented with 2% B27 supplement, 0.4% bovine serum albumin (BSA), 20 ng/mL basic fibroblast growth factor (bFGF) and 20 ng/mL epidermal growth factor (EGF) (cat.no.P5453and P5552; Beyotime Institute of Biotechnology, Shanghai, China). Cells were then added in 96-well low attachment plates (Corning Life Sciences, Bedford, MA, USA; cat.no.[CLS3474](https://www.sigmaaldrich.com/catalog/product/sigma/cls3474?lang=zh&region=CN)). The tumor spheres may be observed under the microscope in a week. For passage culture, PC9-BrM cells were added in low attachment 10 cm dish.

**Cancer stem cells isolation**MACS method was employed to collect cancer stem cells. Then, 5×10^7^ cells were fixed in 1 ml MACS buffer and added with DNase for 10 min at 37℃. Cells were then suspended in 100 ml of MACS buffer and subsequently incubated with biotin-conjugated anti-CD24, APC-conjugated antiCD44 and biotin-conjugated anti-ESA antibodies followed by incubation with anti-biotin antibody and anti-APC antibodies at room temperature for 10 min. CD24^-^, CD44^+^ and ESA^+^ fractions were collected by serial passages through magnetic columns according to the manufacturer’s protocol.

**Animal experiments**

To establish the brain metastasis NSCLC cell line, female BALB/c nude mice (4 weeks old) were purchased from Vital River Laboratory Animal Technology (Beijing, China). The mice were placed in an anesthesia induction box and anesthetized with isoflurane (induced concentration, 3-4%) for 2-3 min and maintained anesthetized using at 1-1.5% isoflurane. The nude mice were inoculated with 5×10^5^ luciferase-labeled PC9/H1915 cells in PBS (100 µL) into left cardiac ventricle. After 65-75 days, the brain metastasis of cancer cells was examined by Bruker In Vivo system. Then all the mice were sacrificed using CO_2_ (30% volume displacement per min) and the brain were removed in sterile conditions. Subsequently the brain was minced and cultured in RPMI-1640 medium. This selection was performed twice.

To obtain a highly and specific brain metastatic mice model, we developed a cancer stem cell (CSCs) derived brain metastasis model in mice. Then the nude mice (n=10) were injected with luciferase-labeled PC9-BrM cell or PC9-BrM/CSCs in PBS (5×10^5^, 100 µL) into left cardiac ventricle. The mice were placed in an anesthesia induction box and anesthetized with isoflurane (induced concentration, 3-4%) for 2-3 min and maintained anesthetized using at 1-1.5% isoflurane. The PC9-BrM/CSCs cells (luciferase-labeled) were fixed in 0.1 mL BD Growth Factor Reduced Matrigel (Becton Dickinson Company, Tokyo, Japan; cat.no.354230) with DMEM medium (5:3). Firefly luciferase-labeled cells were generated by lentivirus infection (GeneChem, Shanghai, China).

The brain metastasis development was detected and the bioluminescence imaging (photons/s/cm^2^/sr) was measured using Bruker In-Vivo FX PRO Imaging System (Bruker corporation, Germany). After 50 days or present the signs of morbidity, all mice were sacrificed using CO_2_ (30% volume displacement per min) and the brain were removed and fixed in 4% paraformaldehyde. The histologically analysis was conducted by H-E staining. Approximately 100 sections (10 mm) were prepared and stained with hematoxylin and eosin. All applicable international, national and/or institutional guidelines or the care and use of animals were followed. These procedures were performed in accordance with the Declaration of Helsinki and approval by the Institutional Review Board of Harbin Medical University .

**Statistical analysis**

All data were presented as mean ± SD based on at least three independent experiments. Statistical analysis was analyzed using SPSS 20.0 (IBM Corp.). Comparisons between groups were examined by One-way ANOVA with post hoc Tukey’s test or Student’s t-test. The overall survival was assessed with Kaplan-Meier plots and log-rank test using GraphPad Prism 5.0 (GraphPad Software, Inc., La Jolla, CA, USA). P<0.05 was regarded as statistically significant.

Results

Establishment of brain metastasis NSCLC cell lines

To investigate the effect of miR-596-3p on the tumor metastasis process, we employed PC9 cell, a NSCLC cell line isolated from lymph node-derived human lung adenocarcinoma cell lines [25] and the H1915 cells, which was probably derived from a lung cancer patient with brain metastasis according to the description in the ATCC [26] to develop a new brain high-metastatic cell line. The PC9 and H1915 cells were firstly inoculated into BALB/c nude mice by intracardiac injections to isolate sub-populations of cells that colonized in the brain for two cycles of selection. (Fig. 1A). The brain metastasis (BrM) cell lines may metastasize to some organs other than the brain, like lung, bone and liver. Brain metastatic lesion was monitored by bioluminescence imaging by employing luciferin injections (intraperitoneal, Fig. 1B).

After tumour dissociation and cell passage in culture, the sub-populations were conducted to a second cycle of *in vivo* selection, yielding brain metastatic derivative cell populations (PC9-BrM and H1915-BrM), which displayed an increased capacity of brain metastatic compared with the primary cell line [27]. We identified that PC9-BrM and H1915-BrM cells presented morphological characters of the epithelial-mesenchymal transition (EMT) such as elongated and fibroblast-like morphology (Fig. 1C). We then detected the expression of EMT related biomarkers, the results indicated that PC9-BrM and H1915-BrM cells undergo EMT process (Fig. 1D). Subsequently, we investigated the invasive capacity of PC9-BrM and H1915-BrM cells and the results suggested a higher invasiveness compared with parental NSCLC cells than parental PPC9 and H1915 cells (Fig. 1E). These results revealed that PC9-BrM and H1915-BrM cells presented mesenchymal features and significantly elevated the invasiveness.

The expression of miR-596-3p is decreased in brain metastatic lesions of NSLC cancer patients

In order to investigate the key microRNAs that are differentially expressed in the progression process of brain metastaisis, we collected the microRNA data in NSCLC cell line (PC-9), Brain metastatic derivative cells (PC-9 BrM) and PC-9 parental cell by extracting the expression profiling data from the Gene Expression Omnibus from the GEO database (GSE83132). The edge R package of R software was used to determine differentially expressed miRNA with significant differences (fold change>2 and false discovery rate (FDR)<0.01) based on the Benjamini-Hochberg method [24]. It turned out that a series of miRNAs was altered in PC-9 BrM compared with that in PC-9 parental cells and top 20 differentially expressed miRNAs were displayed (Fig. 2A and B).

In this study, we also extracted the total RNA from the clinical specimens of primary tumors and paired metastatic tumors in the brain, followed by the Affymatrix GeneChip miRNA 1.0 Array. These data showed that the expression of 10 miRNAs were significantly increased while ten miRNAs were decreased in brain metastatic lesions (Fig. 2C, fold change (FC)>2 and false discovery rate (FDR)<0.01). By overlapping these data, we found that miR-596-3p was significantly downregulated in the metastatic brain tumors and brain metastasis cells compared to the primary NSCLC tissues/cells. To confirm this observation, we subsequently explored the primary tumors from NSCLC patients with or without brain metastasis and also in tumor from brain metastatic lesions. As shown in Fig. 2D, the expression of miR-596-3p was significantly reduced in the brain metastatic lesions, indicating a potential role of miR-596-3p in brain metastasis.

Integrative analysis for the identification of genes involved in NSCLC

Given the lack of early diagnosis and effective therapies of NSCLC at the early stages, it is vital to explore the underlying mechanism. Hence, we designed a comprehensive, cross-platform integrated analysis of NSCLC. We examined the level of miR-596-3p from the blood of healthy donors and early stage NSCLC patients. Notably, a significantly higher level of miR-596-3p was determined in the circulation of healthy donors (n=30),the patients with early stage of NSCLC (n=30) and brain metastasis patients (n=30) (Fig. 3A). In addition, we investigated the level of miR-596-3p in PC9, H1915, A549, H460, PC9-BrM and H1915-BrM cells. The latter two cell lines were generated from PC9 and H1915 cells through multiple rounds of *in vivo* selections of clones that specifically metastasized to brain [27]. We detected that the expression of miR-596-3p was significantly decreased in PC9-BrM and H1915-BrM cells compared to the normal lung cell line (Beas-2B) and parental cells, indicating that miR-596-3p is associated with in the progression of brain metastasis (Fig. 3B).

The miR-596-3p was subjected to GO analyses to identify main functions, and the data were collected from GEO database (https://www.ncbi.nlm.nih.gov/geo/). GO analysis showed that miR-596-3p was related to the development of NSCLC and may affect cell migration and invasion (Fig. 3C). KEGG pathway analysis demonstrated that signaling pathways involved in ECM receptor interaction, cell cycle, Ribosome pathway and p53 signaling pathway were enriched (Fig. 3D). We also performed GSEA (Gene-set enrichment analysis) for the patients with brain metastasis (n=13) and patients without any distant metastasis (n=72) by using top 100 most predicted miR-596-3p target genes based on StarBase V2.0 analysis. Notably, the expressions of miR-596-3p target genes were significantly enriched in the patients with brain metastasis, which further confirm our study that the miR-596-3p is involved in the development of brain metastasis by modulating groups of target genes (Fig. 3E). All these results indicated that miR-596-3p genes are highly likely related to the occurrence of cancer.

Genes of similar categories cluster together In order to investigate the molecular mechanisms of NSCLC, the present study re‑analyzed the gene expression profiles of GSE11969. The interactive web tool, GEO2R (www.ncbi.nlm.nih.gov/geo/geo2r) was applied to screen and identify the differentially expressed genes (DEGs) between normal lung tissue and NSCLC samples. The metastasis related genes which were associated with miR-596-3p (top 100 most predicted) in GSE11969 were investigated. Gene expression levels were correlated pairwise for all candidate genes to identify the potentially common regulatory mechanisms based on concordant or discordant expression patterns (Fig. 3F). We observed strong inter-gene correlations among a majority of cytokine/chemokine genes (interleukins, ILs) and matrix degrading enzymes (metalloproteinases). Therefore, the findings of the present study may provide further understanding of NSCLC development and lead to an improved diagnosis of NSCLC.

miR-596-3p suppresses the expression of YAP1 gene

To investigate the role of miR-596-3p in brain metastasis, we detected the potential target genes in four database (Starbase V2.0, TargetScan, miRanda and miRDB) and identified that six genes were overlapping with a criterion of P-value<0.01 (Fig. 4A). Then the candidate genes were determined in PC9-BrM cells transfected with miR-596-3p or miR-NC by the qRT-PCR assay. The result showed that YAP1 was significantly reduced in transfected PC9-BrM cells (red dots, Fig. 4B, C and Additional Fig.-1A). The potential binding site of YAP1 3’UTR and miR-596-3p are displayed in Fig. 4D. To determine the effect of miR-586-3p on the expression of YAP1, we increased the level of miR-596-3p in PC9-BrM and H1915-BrM cells. The data indicated that YAP1 was upregulated in PC9-BrM and H1915-BrM cells compared with the parental cells and increased the level of miR-596-3p significantly reduced the YAP1 expression (Fig. 4E). However, decreased of miR-596-3p significantly enhanced YAP1 expression in PC9 and H1915 cells (Fig. 4F).

To further verify these results, we co-transfected miR-596-3p mimics or miR-NC with YAP1-WT or YAP1-MUT in 293T cell. Luciferase reporter assays suggested that the reporter activity was decreased after co-transfection of the YAP1-WT and miR-596-3p mimics, while the cells co-transfected with YAP1-MUT and miR-296-3p mimics or miR-NC did not limit the luciferase activity (Fig. 4G). Moreover, we explored the relationship between miR-596-3p and YAP1 in clinical brain metastatic lesions by RT-qPCR assay, and the result showed that miR-596-3p was negatively correlated with YAP1 in brain metastatic samples (Fig. 4H). We also performed meta-analysis of the YAP1 expression by collecting the patients with or without brain metastasis from a combined GEO databases (GSE8894, GSE11969, GSE68465, GSE37745 and GSE13213) and provided evidence that YAP1 was significantly increased in patient with brain metastasis (Fig. 4I). Then YAP1 was subject to GO analyses to identify main functions, and the result showed that YAP1 was related to the process of metastasis and may affect GAP junction and diapedesis (Fig. 4J)

miR-596-3p suppresses trans-endothelial cell migration by blocking YAP1-induced MMP2 expression

To further explore the role of miR-596-3p and YAP1 on NSCLC brain metastasis, we detected the trans-endothelial cell migration capacity of brain metastasis cells by using an *in vitro* blood-brain-barrier (BBB) model, which was established by employing the transwell chamber coated with human brain endothelial cells (HBMEC) and normal human astrocytes (NHA) (Fig. 5A). Then we detected that miR-596-3p significantly inhibited the transmigration ability of PC9-BrM cells, while increase of YAP1 may restrain the suppressive effect of miR-596-3p (Fig. 5B). In this research, we also measured the effect of YAP1 on the trans-endothelial cell migration capacity of PC9 and PC9-BrM cells by using the *in vitro* BBB model. The result showed that overexpression of YAP1 may promote the trans-migratory capacity of PC9-BrM cells than PC9 parental cells (Fig. 5C). Moreover, we conducted the transwell assay to confirm the migratory capacity. The result suggested that overexpression of miR-596-3p significantly suppressed the mobility of PC9-BrM cells, while increased of YAP1 in PC9-BrM cell may impede the invasive phenotype without abrogating the proliferation ability of cell (Fig. 5D and E). It has been reported that MMP-2 plays a vital role in the process of tumor invasion which was regulated by YAP1 [28].

Then we hypothesized that YAP1 may facilitate brain metastasis by inducing MMP-2. By ELISA assay and RT-qPCR assay, we detected that miR-596-3p significantly reduced the expression and secretion of MMP-2 in PC9-BrM cells, while downregulation of miR-596-3p in PC9 cells, increased of YAP1 in PC9 cells or the group of YAP1+miR-596-3p inhibitor may upregulate the expression of MMP-2 (Fig. 5F and G). Furthermore, to examine the clinical effect of MMP-2 in brain metastasis, we detected the relationship between the MMP-2 level and metastatic status of NSLCLC patients by employing the GEO data base that was used in Fig. 4I. We investigated that the level of MMP-2 was significantly correlated with brain and overall metastasis-free survival, indicating that MMP-2 facilitates metastasis in an organ preferential manner (Fig. 5H).

**Suppressing miR-596-3p increases BBB permeability by promoting secretion of IL-8**

The previous section explored that miR-596-3p is capable of impeding trans-endothelial migration, however the effect is partially reserved by increase of YAP1. The result strongly indicates that there may exist other downstream regulators of miR-596-3p, which may affect the BBB permeability. In the therapeutic strategy against the permeability of BBB, tumor-induce inflammatory cytokines were frequently used to increase the permeability [29-30]. Therefore, we made an assumption that miR-596-3p may indirectly modulate the expression of various cytokine by employing the Cytokine Expression Array (Abcam) including 80 cytokines targets. As shown in Fig. 6A, we explored that IL-8 is significantly reduced in the conditioned medium of PC9-BrM cells transfect with miR-596-3p, which was capable to increase the permeability of BBB [31].

We further determined this suppressive role of miR-596-3p on IL-8 in PC9-BrM and H1915-BrM cells by RT-qPCR assay and immunofluorescence assay (Fig. 6B and C). Meanwhile, we decreased miR-596-3p and increased the YAP1 level in miR-596-3p group of PC9 parental cells and explored that the expression of IL-8 was significantly increased (Fig. 6D). To determine whether miR-596-3p inhibits IL-8 by directly targeting with its 3’UTR sites, we employed the IL-8 3’UTR reporter activity. It was found that there was no significant difference on the luciferase assay, suggesting that miR-596-3p may regulate IL-8 level in an indirectly regulatory manner (Fig. 6E). Moreover, to validate the role of IL-8 in facilitating brain metastasis by increasing the penetration of BBB, we conducted trans-endothelial cell migration assay by adding recombinant IL-8 or increasing the level of IL-8 in PC9 cells. The results showed that high level of IL-8 may elevate the trans-migratory capacity of PC9 cells. We also detected the effect of IL-8 on the trans-endothelial cell migration of PC9-BrM cells using the same assay system and found that ectopic expression of IL-8 indeed significantly impeded the transmigration ability of miR-596-3p in PC9-BrM cells. These result suggested that miR-596-3p inhibitor may facilitate the trans-migration of cells by increasing the level of IL-8. (Fig. 6F and G). We also employed the Trans Epithelial Electric Resistance (TEER) assay by using HBMEC cells with recombinant IL-8 or conditioned medium (CM) collected from PC9-BrM cells with miR-596-3p/miR-NC. The data indicated that recombinant IL-8 or CM from PC9-BrM cells reduced the TEER value of HBMEC cells compared to the CM collected from PC9-BrM+miR-596-3p and the control medium (Fig. 6H). These results indicated that miR-596-3p inhibits the capacity of brain metastasis which partially by restraining IL-8-induced BBB permeability.

**miR-596-3p suppresses brain metastasis in a cancer stem cell derived brain metastasis model**

To identify microRNAs which are specific to metastatic CSCs, we first isolated CSCs
population using well established markers, CD24-, CD44+ and ESA+, from NSCLC brain metastasis cells. These cells were explored for their tumor initiating ability by injecting them into mice. The data of our limiting dilution analysis suggested that the isolated CSCs (CD24-/CD44+/ESA+) population has significantly stronger ability of generating tumors compared to non-stem cell population. We then determined the metastatic ability of these cells by implanting CSCs into mice via intracardiac injection.

Subsequently the effect of miR-596-3p in metastatic ability of brain metastatic cells *in vivo* was examined . To obtain a high success rate of brain metastatic mice model, CSCs were collected from PC9-BrM and they were inoculated into nude mice through intracardiac injection (1×10^6^, suspended with Matrigel), followed by monitoring brain metastasis with Bruker In-Vivo system for 90 days. To further explore the role of miR-596-3p in the stemness of PC9-BrM/CSCs cells. We investigated the level and the self-renewal ability of miR-596-3p in PC9-BrM/CSCs cells. Then the western blot assays demonstrated that miR-596-3p decreased the level of specific stemness markers, including Bmi-1, Oct-4, Sox-2 and Nanog (Additional Figure-1C).

As shown in Fig.7A and B, the PC9-BrM/CSCs showed higher rate of brain metastasis and less brain metastasis-free survival time compared with PC9-BrM parental cells. We next examined the effect of miR-596-3p in metastatic ability of CSCs *in vivo*. CSCs were prepared from PC9-BrM that was transfected with lentivirus carrying with or without miR-596-3p. Then we determined the specificity of miR-596-3p expression in BrM/CSCs and non-stem cell population. The generality of this observation was further confirmed by examining the expression of miR-596-3p in PC9 and H1915 cell lines, and their metastatic variants. Then we identified that the expression of miR-596-3p was significantly reduced in the brain metastatic variants of these cells in a CSCs-specific manner (Additional Fig. 1B).

Bioluminescent (BLI) image right after the CSCs inoculation demonstrated distribution of CSCs cells in the whole body suggesting a successful intracardiac injection. After eight weeks, metastatic tumor observed in the brain was prominent in all mice that received CSCs transfected with miR-NC only. On the other hand, mice that injected of CSCs carrying miR-596-3p displayed significantly less metastatic lesions in the brain, suggesting that miR-596-3p may suppress the brain metastasis capacity of PC9-BrM/CSCs (Fig. 7C and D). H&E staining indicated the lesion of microscopic metastasis in the brains of 6/10 mice receiving injections of PC9-BrM/CSCs+miR-596-3p. Ten sections of each brain were employed to calculate the number of micro-metastasis lesions. Mice transfected with miR-596-3p CSCs showed significant decrease in the formation of micrometastatic lesion (Fig. 7E and F). Representative bioluminescent images showed lung caner cells preferentially metastasis to the brain and bone. We observed miR-596-3p/CSCs group mice to have decreased brain metastasis (3/10), but increased bone metastasis (7/10) compared to the miR-NC/CSCs group (brain metastasis, 6/10 and bone metastasis, 4/10). These results suggest that miR-596-3p promotes brain metastasis of circulating breast cancer cells.

Discussion

Metastatic colonization of the brain is the most common and detrimental stage for the lifetime prevalence approaching 25% in NSCLC patients [1]. Brain metastasis may cause significant neurologic diseases, cognitive disorder and emotional difficulties [2]. However, the molecular mechanisms underlying the process of brain metastasis remains obscure. The classical hypothesis of "seed and soil" suggests that metastatic tumor cells could develop microenvironment for their growth and survival through complex interactions. Meanwhile, accumulating evidence implies that epigenetic regulation plays a vital role in tumorigenesis and metastasis [32,33]. In this research, we evaluated the miRNAs from tumor tissues collected from primary NSCLC tumors and paired brain metastatic lesions followed by microRNA profiling, and the paired tissues study (lung cancer and brain metastasis) was the first time used in the process of brain metastasis.

It has been indicated that tumor cells often metastasize to preferred organs and are partly restricted by microRNAs [34]. In the brain metastasis condition, the decrease of miR-596-3p in brain NSCLC samples indicated that the miRNA may be involved in “brain-seeking” metastatic potential,which may be involved in the progression of BrM from primary NSCLC. This hypothesis was supported by our observation that miR-596-3p is downregulated in both primary lung cancer specimens and brain metastasis samples. Wei et al. demonstrated that miR-330-3p promotes brain metastasis and EMT process in non-small cell lung cancer [15]. Singh et al. have recently shown that blocking the STAT3-miR-21 axis may form a strong notion for a potential therapeutic strategy in patients with lung-to-brain metastasis [35]. Sun et al. [14] have suggested that miR-4270 downregulation and miR-423-3p upregulation was associated with an increased risk of brain metastasis in NSCLC patients and these miRNAs might be useful prognostic and clinical treatment targets. More recently, it was found that miR-95-3p is decreased in brain metastatic tissues compared with lung adenocarcinoma and may represent a critical role in conferring brain metastatic potential to lung cancer cells [36]. However, the biological function of miR-596-3p has not been sufficiently examined. To our knowledge, there is no previous study about the role of miR-596-3p in lung cancer. These results showed that the microRNA network may exert as a vital role in the metastatic process of lung cancer.

In this study, we explored microRNA profiling on a cohort of 12 pairs of primary samples and brain metastatic tumor tissues from NSCLC patients for the first time in study of NSCLC brain metastasis and investigated the differentially expressed miRNAs in GEO database. By overlapping two set of data, we detected that the miR-596-3p is involved in the formation of brain metastasis by targeting YAP1-induced MMP-2 secretion which regulate the permeability of BBB. Our research further validated that brain metastasis is a highly dynamic multi-stage process and involved a series of key factors which are affected by at least one or few microRNAs. YAP1 acts as an oncogene which promotes the tumor progression by targeting various signaling pathways [37]. Hu et al. detected that overexpression of YAP1 was correlated with ARHGAP29, which may cause cytoskeletal rearrangement and then promote the progression and metastasis in gastric cancer [38]. And overexpression of YAP1 significantly enhanced the brain metastatic ability in less brain metastatic potential NSCLC cell line, indicating that YAP1 may exert the post-transcriptional regulatory in the process of YAP1-mediated brain metastasis. We also noticed that the number of patients included in this study was a significant limitation. but since additional well-annotated samples were not readily available to us. We wish that our study will help other researcher to independently validate our findings in their specimen collections.

Metastasis is a multi-step process which include the intravasation into vessel, survival in the circulation, extravasation and establish colonization in brain parenchyma [39,40]. In this study, we detected that miR-596-3p may reduce the secretion of IL-8 which lead to a suppression of BBB permeability of cancer cells into the brain. Previous studies have indicated that pro-inflammatory cytokines such as TGF-β and IL-8 may impede the penetration of BBB by limiting the cell-cell adhesion proteins [41,42]. Then we detected the role of miR-596-3p on IL-8 using the luciferase reporter assay, however the effect is not statistically significant, indicating that the effect is indirect. In this study, we have shown that miR-596-3p suppressed the level of IL-8 which in turn decreased the BBB permeability and the penetration of cancer cells into the brain. Several studies have previously shown that pro-inflammatory cytokines such as TNFα and IL-8 were able to alter the permeability of BBB by affecting the tight junction proteins [43-45]. These results are consistent with the data that miR-596-3p mediated IL-8 plays a critical role in brain metastasis of NSCLC cells.

How miR-596-3p modulates IL-8 expression is an intriguing question. When we tested the effect of miR-596-3p on TNFα using a 3’UTR reporter plasmid, we did not see any effect of miR-596-3p (Fig-7E), suggesting that the effect is indirect. We then detected the transcription factors that potentially control IL-8 expression by conducting the human transcription factor ChIP sequence data which was released by the ENCODE project [46-47]. We found several such factors; however, none of them were potential targets of miR-596-3p based on our Targetscan analysis. Therefore, the underlying mechanism of miR-596-3p modulating IL-8 expression is unknown, which may be involved in the regulatory network of brain metastasis process.

Xenograft models constructed by intracardiac injection of human tumor cells into immunodeficient mice have been regarded as useful tools for the study of metastasis. However, the success rate of xenograft tumor model established by anchorage-dependent cell is not high. Recently, cancer stem-like cells (CSCs) are considered to play a role in metastatic progression of cancer and that these cells may exist in tumor mass even at an early stage. These findings led us to construct a NSCLC/CSCs derived brain metastasis model. In addition, the formation and success rate of brain metastases model is higher than that of anchorage-dependent cell intracardiac injection models. The CSCs derived model may screen the cell population of high proliferation rate and self-renewal, which may better form the brain metastasis model. The model formation cycle and successful rate may also be improved. This brain metastasis model acquired the same character of NSCLC patients and the feature of brain metastatic potential. And the CSCs derived brain metastasis model acquired the same brain metastasis capacity compared with the anchorage-dependent cell model, which was confirmed in this study. Therefore, our cancer stem cell derived model of NSCLC metastasis provided an effective strategy for the study of NSCLC development.

In conclusion, our study suggested that miR-596-3p/YAP1 pathway may provide a promising therapeutic strategy for brain metastasis of NSCLC, which may mitigate brain metastasis development and behavior, leading to an improvement of prognosis for patients with metastatic brain cancer.

**DATA AVAILABILITY STATEMENT**The data that support the findings of this study are available from the corresponding author upon reasonable request.

**ACKNOWLEDGEMENTS**

The present study was supported by Haiyan Fund Project of Harbin Medical University Cancer Hospital (grant no. JJMS2021-08), Heilongjiang Provincial Postdoctoral Science Foundation (grant no. LBH-Z20073), Natural Science Foundation of Heilongjiang Province (grant no. JJ2021LH1458), Postdoctoral Science Foundation of China (grant no. 2021M693819) and the King Abdullah University of Science and Technology (KAUST) Office of Sponsored Research (OSR) under Award No. URF/1/3450-01-01, URF/1/4098-01-01, REI/1/4216-01-01, REI/1/4437-01-01, and REI/1/4473-01-01. The authors acknowledge and appreciate colleagues for valuable efforts and comments on this paper.

**Conflict of Interest Statement**

The authors declare no conflicts of interest.

**AUTHOR CONTRIBUTIONS**

C. L., H. Z., Y. H. , H. J. and J. X. designed and performed the experiments, analyzed and interpreted data, and C. L. and H. L. wrote the manuscript; Y. .H., S. A. and Y. W. provided technical and material support, and T. S. , H. J. and G. S. acquisition and analyzed data; X. Xiao, T. F. and Y. W. edited the manuscript; P. L. and G. X. designed experiments and critically reviewed the paper.

**ETHICS STATEMENT**Animal procedures were approved via the Institutional Animal Care and Use
Committee of Harbin Medical University and were processed in accordance with the guideline of Animal Care and Use Committee of the National Institutes of Health.

**References**

1. Eichler AF, Loeffler JS. Multidisciplinary management of brain metastases. Oncologist. 2007;12:884-898.
2. Soffetti R, Ruda R, Mutani R. Management of brain metastases. J Neurol. 2002;249:1357-1369.
3. Schouten LJ, Rutten J, Huveneers HA, Twijnstra A. Incidence of brain metastases in a cohort of patients with carcinoma of the breast, colon, kidney, and lung and melanoma. Cancer. 2002;94:2698-2705.
4. [Hanna N](https://www.ncbi.nlm.nih.gov/pubmed/?term=Hanna N[Author]&cauthor=true&cauthor_uid=15117980), [Shepherd FA](https://www.ncbi.nlm.nih.gov/pubmed/?term=Shepherd FA[Author]&cauthor=true&cauthor_uid=15117980), [Fossella FV](https://www.ncbi.nlm.nih.gov/pubmed/?term=Fossella FV[Author]&cauthor=true&cauthor_uid=15117980), [Pereira JR](https://www.ncbi.nlm.nih.gov/pubmed/?term=Pereira JR[Author]&cauthor=true&cauthor_uid=15117980), [De Marinis F](https://www.ncbi.nlm.nih.gov/pubmed/?term=De Marinis F[Author]&cauthor=true&cauthor_uid=15117980), [von Pawel J](https://www.ncbi.nlm.nih.gov/pubmed/?term=von Pawel J[Author]&cauthor=true&cauthor_uid=15117980), et al. Randomized phase III trial of pemetrexed versus docetaxel in patients with non-small-cell lung cancer previously treated with chemotherapy. J Clin Oncol. 2004;22:1589-1597.
5. Villa S, Weber DC, Moretones C, Manes A, Combescure C, Jove J, et al. Validation of the new Graded Prognostic Assessment scale for brain metastases: a multicenter prospective study. Radiat Oncol. 2011;6:1-8.
6. [Ge M](https://www.ncbi.nlm.nih.gov/pubmed/?term=Ge M[Author]&cauthor=true&cauthor_uid=28780743), [Zhuang Y](https://www.ncbi.nlm.nih.gov/pubmed/?term=Zhuang Y[Author]&cauthor=true&cauthor_uid=28780743), [Zhou X](https://www.ncbi.nlm.nih.gov/pubmed/?term=Zhou X[Author]&cauthor=true&cauthor_uid=28780743), [Huang R](https://www.ncbi.nlm.nih.gov/pubmed/?term=Huang R[Author]&cauthor=true&cauthor_uid=28780743), [Liang X](https://www.ncbi.nlm.nih.gov/pubmed/?term=Liang X[Author]&cauthor=true&cauthor_uid=28780743), [Zhan Q](https://www.ncbi.nlm.nih.gov/pubmed/?term=Zhan Q[Author]&cauthor=true&cauthor_uid=28780743). High probability and frequency of EGFR mutations in non-small cell lung cancer with brain metastases. [J Neurooncol](https://www.ncbi.nlm.nih.gov/pubmed/?term=Ge+M,+Zhuang+Y,+Zhou+X,+et+al.+High+probability+and+frequency+of+EGFR+mutations+in+non-small+cell+lung+cancer+with+brain+metastases[J].+Journal+of+Neuro-Oncology,+2017(7):1-6.). 2017;135:413-418.
7. Steeg PS, Camphausen KA, Smith QR. Brain metastases as preventive and therapeutic targets. Nat Rev Cancer. 2011;11:352–363.
8. Arslan C, Dizdar O, Altundag K. Systemic treatment in breast-cancer patients with brain metastasis. Expert Opin Pharmacother. 2010; 11:1089–1100.
9. Xiao J, Chen L, Mao GS, Gao WY, Lu M, He XJ, Huang HY. Neurorestorative clinical application standards for the culture and quality control of olfactory ensheathing cells. J Neurorestoratology, 2017;5: 175-179.
10. Fidler IJ. The role of the organ microenvironment in brain metastasis. Semin Cancer Biol. 2011;21:107-12.
11. Berkhout B, Jeang KT. RISCy business: microRNAs, pathogenesis, and viruses. J Biol Chem. 2007;282:26641-26645.
12. Yu SL, Chen HY, Chang GC, Chen CY, Chen HW, Singh S, et al. MicroRNA signature predicts survival and relapse in lung cancer. Cancer Cell. 2008;13:48-57.
13. Volinia S, Calin GA, Liu CG, Ambs S, Cimmino A, Petrocca F, et al. A microRNA expression signature of human solid tumors defines cancer gene targets. Proc Natl Acad Sci USA. 2006;103:2257-2261.
14. Sun G, Ding X, Bi N, Wang Z, Wu L, Zhou W, et al. Molecular predictors of brain metastasis-related microRNAs in lung adenocarcinoma. PLoS Genetics. 2019; 2:e1007888.
15. Wei C, Zhang R, Cai Q, Gao X, Tong F, Dong J, et al. MicroRNA-330-3p promotes brain metastasis and epithelial-mesenchymal transition via GRIA3 in non-small cell lung cancer. Aging. 2019; 17:6734-6761.
16. Sun G, Ding X, Bi N, Wu L, Wang J, Zhang W, et al. MiR-423-5p in brain metastasis: potential role in diagnostics and molecular biology. Cell Death Dis. 2018; 10:936.
17. J Remon, D Alvarez-Berdugo, M Majem, T Moran, N Reguart, P Lianes. miRNA-197 and miRNA-184 are associated with brain metastasis in EGFR-mutant lung cancers. Clin Transl Oncol. 2016; 2:153-159.
18. Liu CG, Calin GA, Volinia S, Croce CM. MicroRNA expression profiling using microarrays. Nat Protoc 2008;3:563-78.
19. McDermott R, Gabikian P, Sarvaiya P, Ulasov I, Lesniak MS. MicroRNAs in
    brain metastases: big things come in small packages. J Mol Med. 2013;91:5-13
20. Alsidawi S, Malek E, Driscoll JJ. MicroRNAs in brain metastases: potential role as diagnostics and therapeutics. Int J Mol Sci. 2014;15:10508-26.
21. D’Addario G, Fruh M, Reck M, Baumann P, Klepetko W, Felip E, et al. Metastatic non-small-cell lung cancer: ESMO Clinical Practice Guidelines for diagnosis, treatment and follow-up. Ann Oncol. 2010;21:192-237.
22. Bos PD, Zhang XH, Nadal C, Shu W, Gomis RR, Nguyen DX, et al. Genes that mediate breast cancer metastasis to the brain. Nature. 2009;459:1005-1009.
23. Hawkins SF, Guest PC. Multiplex Analyses Using Real-Time Quantitative PCR. Methods Mol Biol. 2017;1546:125-133.
24. Benjamini Y, Hochberg Y. Controlling The False Discovery Rate-A Practical And Powerful Approach To Multiple Testing. [J R Stat Soc](http://www.medsci.cn/sci/submit.do?id=4eae4264). 1995;1:289-300.
25. Nguyen DX, Chiang AC, Zhang XH, Kim JY, Kris MG, Ladanyi M, et al. WNT/TCF signaling through LEF1 and HOXB9 mediates lung adenocarcinoma metastasis. Cell. 2009;138:51-62.
26. Nolte SM, Venugopal C, McFarlane N, Morozova O, Hallett RM, O’Farrell E, et al. A cancer stem cell model for studying brain metastases from primary lung cancer. J Natl Cancer Inst. 2013;105:551-562.
27. Tominaga N , Kosaka N , Ono M , Katsuda T, Yoshioka Y, Tamura K, et al. Brain metastatic cancer cells release microRNA-181c-containing extracellular vesicles capable of destructing blood–brain barrier. Nat Commun. 2015;6:6716.
28. Zhang J , Xu ZP , Yang YC , Zhu JS, Zhou Z, Chen WX. Expression of Yes-associated protein in gastric adenocarcinoma and inhibitory effects of its knockdown on gastric cancer cell proliferation and metastasis. Int J Immunopathol Pharmacol. 2012; 25:583.
29. Benveniste EN. Inflammatory cytokines within the central nervous system: sources, function, and mechanism of action. Am J Physiol. 1992;263:C1-C16.
30. D. Lucas SM, Rothwell NJ, Gibson RM. The role of inflammation in CNS injury and disease. Br J Pharmacol. 2006;147(Suppl 1):S232-S240.
31. E.Tsao N, Hsu HP, Wu CM, Liu CC, Lei HY. Tumour necrosis factor-alpha causes an increase in blood-brain barrier permeability during sepsis. J Med Microbiol. 2001;50:812-821
32. [Valastyan S](https://www.ncbi.nlm.nih.gov/pubmed/?term=Valastyan S[Author]&cauthor=true&cauthor_uid=19524507), [Reinhardt F](https://www.ncbi.nlm.nih.gov/pubmed/?term=Reinhardt F[Author]&cauthor=true&cauthor_uid=19524507), [Benaich N](https://www.ncbi.nlm.nih.gov/pubmed/?term=Benaich N[Author]&cauthor=true&cauthor_uid=19524507), [Calogrias D](https://www.ncbi.nlm.nih.gov/pubmed/?term=Calogrias D[Author]&cauthor=true&cauthor_uid=19524507), [Szász AM](https://www.ncbi.nlm.nih.gov/pubmed/?term=Szász AM[Author]&cauthor=true&cauthor_uid=19524507), [Wang ZC](https://www.ncbi.nlm.nih.gov/pubmed/?term=Wang ZC[Author]&cauthor=true&cauthor_uid=19524507), et al. A pleiotropically acting microRNA, miR-31, inhibits breast cancer metastasis. Cell. 2009;137:1032-1046.
33. [Wood SL](https://www.ncbi.nlm.nih.gov/pubmed/?term=Wood SL[Author]&cauthor=true&cauthor_uid=24176790), [Pernemalm M](https://www.ncbi.nlm.nih.gov/pubmed/?term=Pernemalm M[Author]&cauthor=true&cauthor_uid=24176790), [Crosbie PA](https://www.ncbi.nlm.nih.gov/pubmed/?term=Crosbie PA[Author]&cauthor=true&cauthor_uid=24176790), [Whetton AD](https://www.ncbi.nlm.nih.gov/pubmed/?term=Whetton AD[Author]&cauthor=true&cauthor_uid=24176790). The role of the tumor-microenvironment in lung cancer-metastasis and its relationship to potential therapeutic targets. [Cancer Treat Rev](https://www.ncbi.nlm.nih.gov/pubmed/24176790). 2014;40:558-566.
34. Ramakrishna R, Rostomily R. Seed, soil, and beyond: The basic biology of brain metastasis. Surg Neurol Int. 2013; 4:S256-S264
35. Singh M , Garg N , Venugopal C, Hallett R, Tokar T, McFarlane N, et al. STAT3 pathway regulates lung-derived brain metastasis initiating cell capacity through miR-21 activation. Oncotarget. 2015; 6:27461-27477.
36. Hwang SJ, Lee HW, Kim HR, Song HJ, Lee DH, Lee H, et al. Overexpression of microRNA-95-3p suppresses brain metastasis of lung adenocarcinoma through downregulation of cyclin D1. Oncotarget, 2015;6:20434-20448.
37. Totaro A, Panciera T, Piccolo S. YAP/TAZ upstream signals and downstream responses. Nature Cell Biol. 2018;20:888-899.
38. Qiao Y, Chen J, Lim YB, Finch-Edmondson ML, Seshachalam VP, Qin L, et al. YAP Regulates Actin Dynamics through ARHGAP29 and Promotes Metastasis. Cell Rep. 2017;19:1495-1502.
39. Pantel K, Brakenhoff RH. Dissecting the metastatic cascade. Nat Rev Cancer. 2004;4:448-456.
40. Izraely S, Sagi-Assif O, Klein A, Meshel T, Tsarfaty G, Pasmanik-Chor M, et al. The metastatic microenvironment: brain-residing melanoma metastasis and dormant micrometastasis. Int J Cancer. 2012;131:1071-1082.
41. Yu H, Huang X, Ma Y, Gao M, Wang O, Gao T, et al. Interleukin-8 Regulates Endothelial Permeability by Down-regulation of Tight Junction but not Dependent on Integrins Induced Focal Adhesions. Int J Biol Sci. 2013;9:966-979.
42. Ganatra D , Vasavada A , Vidya N , Gajjar D, Rajkumar S. Trichostatin A Restores Expression of Adherens and Tight Junction Proteins during Transforming Growth Factor β-Mediated Epithelial-to-Mesenchymal Transition. J Ophthalmic Vis Res. 2018;13:274-283.
43. Wang W, Lv S, Zhou Y, Fu J, Li C, Liu P. Tumor necrosis factor-alpha affects blood-brain barrier permeability in acetaminophen-induced acute liver failure. Eur J Gastroenterol Hepatol. 2011; 23:552-558.
44. Yang GY, Gong C, Qin Z, Liu XH, Lorris Betz A. Tumor necrosis factor alpha expression produces increased blood-brain barrier permeability following temporary focal cerebral ischemia in mice. Brain Res Mol Brain Res. 1999; 69:135-143.
45. Saija A, Princi P, Lanza M, Scalese M, Aramnejad E, De Sarro A. Systemic cytokine administration can affect blood-brain barrier permeability in the rat. Life Sci. 1995; 56:775-784.
46. Gerstein MB, Kundaje A, Hariharan M, Landt SG, Yan KK, Cheng C, et al. Architecture of the human regulatory network derived from ENCODE data. Nature. 2012; 489:91-100.
47. Wang XY, Sun N, Meng XQ, Chen M, Jiang CL, Cai JQ. [Review of clinical nerve repair strategies for neurorestoration of central nervous system tumor damage](http://jnr.tsinghuajournals.com/EN/10.26599/JNR.2020.9040018). *J Neurorestoratology*. 2020; 8:172-181.

Figure legend

Fig. 1 **Establishment of brain metastasis NSCLC cell lines. A** Schematic representation of the route for the selected of brain metastatic derivatives in mice. PC9 and H1915 NSCLC cell lines (5×10^5^ cells) were inoculated intracardially into BALB/c nude mice. After 65-75 days, the brain metastasis of cancer cells were explored by *in vivo* bioluminescence imaging system (Bruker In Vivo). The brain-metastasized cancer cells were collected and cultured for another 30 days with Zeocin (50 mg/mL). The selection process was conducted twice, and we collected the cell lines as PC9-BrM and H1915-BrM. **B** The bioluminescence image of mice with the PC9-BrM and H1915-BrM cell brain metastasis. **C** Morphological difference between parental PC9 and H1915 cells and brain metastatic PC9-BrM and H1915-BrM cells was displayed a light microscope (Nikon Corporation) at ×100 or ×200 magnification. **D** To detect the metastatic capacity of cancer cell, EMT-related genes were measured between parental cell and brain metastasis cells. The protein level of EMT-related biomarkers (E-cadherin and Vimentin) was examined by the western blot assay. **E** The invasive ability of PC9/H1915 and PC9-BrM/H1915-BrM cells were validated by a Transwell assay. Data were presented as the means ± standard error of the mean of at least three independent experiments. ^**^*P*<0.01 and ^***^*P*<0.001.

Fig. 2 **miR-596-3p is down-regulated in NSCLC cancer patients with brain metastasis. A** The significantly differentially-expressed miRNA were detected in GEO datasets (GSE83132). **B** Scatter plots suggested the miRNA expression variation between the two compared groups. **C** A heatmap for the microRNAs was generated to analyze the primary tumor (NSCLC) tissues and paired metastatic brain tumors by the microRNA PCR array. The mRNAs were isolated from the formalin-fixed paraffin embedded (FFPE) NSCLC primary tissues (n=5) and paired brain metastatic lesions (n=5). The fold change (FC)>2 and false discovery rate (FDR)<0.01. **D** The expression of miR-596-3p was confirmed by RT-qPCR for primary NSCLC tumors with or without brain metastasis (n=12) and brain metastatic lesions (n=12). Data were presented as the means ± standard error of the mean of at least three independent experiments. ^**^*P*<0.01 and ^***^*P*<0.001.

Fig. 3 **miR-596-3p was explored as a downregulated gene in NSCLC tissues by integrative analysis.** **A** High level of miR-596-3p was detected in the circulation of healthy donors (n=30) compared to the patients with early stage of NSCLC (n=30). **B** The expression of miR-596-3p was examined by RT-qPCR for Beas-2B, A549, H460, PC9, H1915, PC9-BrM and H1915-BrM cells. **C** GO analysis were performed to detect the function of miR-596-3p in cell processes. **D** Enriched KEGG pathway analysis of miR-596-3p associated genes by DAVID. **E** The target genes of miR-596-3p were selected from the top 60 genes predicted by StarBase V2.0 database followed by GSEA (Gene Set Enrichment Analysis) between patients with or without brain metastasis in GSE11969 dataset. **F** Inter-relations of gene expression levels. The gene expression values were conducted to separate different categories for their interrelationship. For this, hierarchical cluster dendrogram was explored using Ward Minimum Variance Method. (In the heat map, the red color shows a strong positive expression correlation while blue color depicts weak expression correlation while the rest color codes fall in between). Data were presented as the means ± standard error of the mean of at least three independent experiments. ^*^*P*<0.05 and ^***^*P*<0.001.

Fig. 4 **miR-596-3p directly targets YAP1.** **A** Venn intersection analysis showed that 6 target genes were commonly investigated by all four databases. **B** miR-596-3p level was determined in PC9-BrM and H1915-BrM cells transfected with miRNA mimics/miR-NC or inhibitor/NC inhibitor by RT-qPCR. **C** Expression of the potential binding mRNAs was elevated by RT-qPCR following transfection with increased miR-596-3p in PC9-BrM cells. **D** The potential target site of YAP1 on miR-596-3p. **E** PC9-BrM and H1915-BrM cells were transfected with miR-596-3p mimics or miR-NC only, and the expression of YAP1 was detected by Western blot. **F** PC9 and H1915 cells were transfected with miR-596-3p inhibitor (50μM) or miR-inhibitor NC, and the expression of YAP1 was examined by Western blot. **G** miR-596-3p could downregulate the luciferase activity. **H** Correlation between miR-596-3p and YAP1 levels was examined (n=12). **I** mRNA expression was determined between the primary NSCLC tissues with or without brain metastasis in a combined GEO databases (GSE8894, GSE11969, GSE68465, and GSE12280). **J** GO analysis was performed to detect the function of YAP1 in metastasis processes. Data were presented as the means ± standard error of the mean of at least three independent experiments. ^*^*P*<0.05, ^**^*P*<0.01 and ^***^*P*<0.001.

Fig. 5 **miR-596-3p suppresses the metastatic ability of PC9-BrM by inhibiting YAP1-mediated MMP-2 expression. A** A schematic diagram of in vitro BBB model. **B** PC9-BrM cells transfected with the miR-596-3p mimics, or both miR-596-3p mimics and YAP1 were cultured on the up chamber pre-coated with normal human astrocytes (NHA) and Human brain Microvascular endothelial cells (HBMEC) on both sides, and the migrated cells was measured after 24 h. **C** PC9 and PC9-BrM cells with the transfected with YAP1 were added on the up chamber and the number of invaded cells was measured after 24 h. **D** Transwell assay for PC9-BrM cells transfected with the miR-596-3p, or both miR-596-3p and YAP1 was performed. **E** CCK-8 assay for PC9-BrM cells transfected with the miR-596-3p, or both miR-596-3p and YAP1 was conducted. **F** MMP-2 expression was detected in PC9-BrM cells and PC9 cells that were transfected with miR-596-3p mimics or miR-inhibitor+YAP1 by ELISA. **G** MMP-2 mRNA expression was explored in PC9 cells that were transfected with miR-596-3p inhibitor/miR-inhibitor NC or pcDNA3.1-YAP1/vector or miR-inhibitor+YAP1 by qRT-PCR. **H** Kaplan-Meier analysis for brain metastasis-free survival of NSCLC patients using a combined GEO databases. Patients were divided into two groups based on the expression status of MMP-2 in their primary tumors. Data were presented as the means ± standard error of the mean of at least three independent experiments. ^*^*P*<0.05, ^**^*P*<0.01 and ^***^*P*<0.001.

Fig. 6 **miR-596-3p suppresses brain metastasis by targeting IL-8.** **A** Conditioned medium from PC9-BrM cells that were transfected with miR-596-3p/miR-NC were conducted by cytokine array (Abcam) analysis. Fold change (FC) was examined by using Image J software. **B** PC9-BrM and H1915-BrM cells were transfected with miR-596-3p/miR-NC, and the expression of IL-8 was determined by qRT-PCR assay. **C** PC9-BrM cell was transfected with miR-596-3p, and the level of IL-8 was detected by immunofluorescence assay. **D** PC9-BrM cells was treated with miR-596-3p inhibitor, miR-inhibitor NC or miR inhibitor+YAP1, and the level of IL-8 was detected by qRT-PCR. **E** Analysis of the luciferase activity of 293T cell co-transfected with miR-596-3p mimics/miR-NC and the luciferase reporter psi-CHECK2-YAP1-WT/MUT. **F** PC9 parental cells were cultured on the upper side of a transwell chamber by adding with various doses of recombinant IL-8, and the number of invaded cells was calculated after 24h. **G** PC9 cells with increased IL-8 were added into the transwell insert, and the number of invaded cells was calculated after 24h. **H** HBMEC cells were added with conditioned medium from PC9-BrM, 231BrM+miR-596-3p and recombinant IL-8, the trans-endothelial electrical resistance was detected by EVOM2 epithelial voltohmmeter.

Fig. 7 **miR-596-3p suppresses brain metastasis *in vivo*.** **A** PC9-BrM transfected with miR-596-3p/miR-NC were grown as tumor-spheres in tumor sphere media. A hallmark of cancer stem cells (CSCs), was validated though assessment of secondary sphere-forming capacity. **B** Kaplan-Meier analysis for brain metastasis-free survival of mice that were injected with PC9-BrM/PC9-BrM-CSCs. **C** PC9-BrM stem cells transfected with miR-596-3p/miR-NC were intracardially injected into nude mice (n=8) followed by monitoring tumor growth by detecting the total bioluminescence in the brain. The line chart indicated the tumor BLI value. **D** Kaplan-Meier analysis was performed for brain metastasis-free survival of mice. **E** The brain histology image of mice injected with miR-596-3p/miR-NC in PC9-BrM-CSCs. **F** Evaluation of micrometastatic lesions in mice brain.

**Additional Fig. 1** **A** Expression of the potential binding mRNAs was elevated by RT-qPCR following transfection with increased miR-596-3p in PC9-BrM cells. **B** The expression of miR-596-3p in BrM/CSCs and non-stem cell population. **C**  Overexpression of miR-596-3p reduced Bmi-1, Oct-4, Sox-2 and Nanog expression, as detected by Western blot assays. The protein level was displayed with bar graph. Data were presented as the means ± standard error of the mean of at least three independent experiments. ^*^*P*<0.05, ^**^*P*<0.01 and ^***^*P*<0.001.
