# Supplementary figures and images for: miR-596-3p suppresses brain metastasis of non-small cell lung cancer by modulating YAP1 and IL-8
Source: Cell Death Dis. 2022 Aug 12;13(8):699. doi: 10.1038/s41419-022-05062-7 (PMC9374706; doi:10.1038/s41419-022-05062-7)

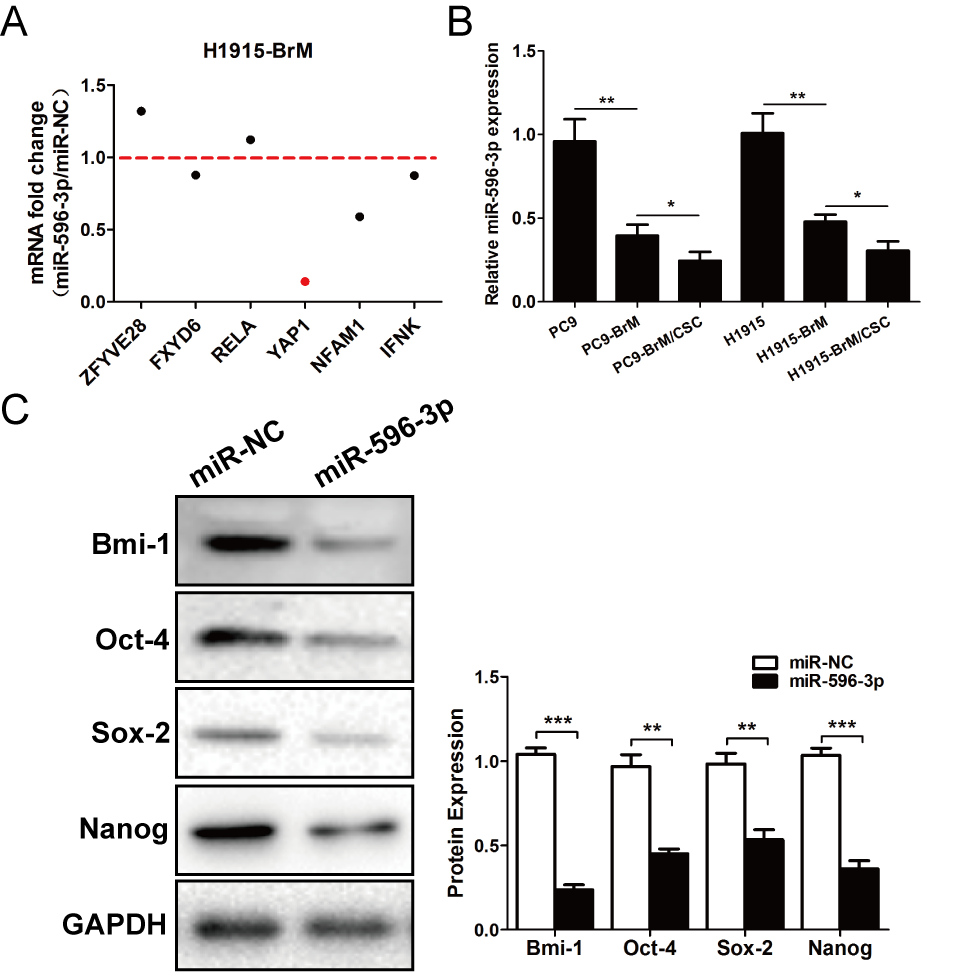

Supplement: Supplementary file 5 — Additional Figure-1 [file 41419_2022_5062_MOESM5_ESM.tif]
