# Supplementary material for: miR-596-3p suppresses brain metastasis of non-small cell lung cancer by modulating YAP1 and IL-8
Source: Cell Death Dis. 2022 Aug 12;13(8):699. doi: 10.1038/s41419-022-05062-7 (PMC9374706; doi:10.1038/s41419-022-05062-7)
Supplement: Supplementary file 1 — Author Declaration [file 41419_2022_5062_MOESM1_ESM.pdf]

AUTHOR DECLARATION

The reference: CDDIS-21-3128R
Title: miR-596-3p suppresses brain metastasis of non-small cell lung cancer by modulating YAP1 and IL-8
By: Chenlong Li

Dear Editor,

Sorry for disturbing you. I am not sure if it is the right time to contact you to inquire about the change in author list. Firstly, during the revision process of this article, we ask a lot help from Dr. Yuxin Huang. She help Dr. Hua Jin finished the work and provided the data of supplement part during the revision stage. Then we planed to remove Dr. Yuxin Huang to the second author of this article. Secondly, we plan to remove Dr. Hua Jin to the fourth author because of his work. And we have got their permission and approved by all named authors in our group.

We have confirmed that the agreement and changes have been read and approved by all named authors.

The new author list:

Chenlong Li[1*], Hongshan Zheng[1*], Jinsheng Xiong[1*], Yuxin Huang[2], Haoyang Li[3,4,5] , Hua Jin[1], Siqi Ai[1], Yingjie Wang[1], Tianqi Su[1], Guiyin Sun[1], Xu Xiao[1], Tianjiao Fu[1], Yujie Wang[1], Xin Gao3[#], Peng Liang[1#]

[1]Department of Neurosurgery, Harbin Medical University Cancer Hospital, Harbin 150001, Heilongjiang, China;
[2]Heilongjiang Tuomeng Technology Co.Ltd., Harbin 150040, Heilongjiang, China.
[3]Computational Bioscience Research Center, King Abdullah University of Science and Technology (KAUST), Thuwal, 23955, Saudi Arabia;
[4]Cancer Systems Biology Center, the China-Japan Union Hospital, Jilin University, Changchun 130001, Jilin, China;
[5]College of Computer Science and Technology, Jilin University, Changchun 130001,

Jilin, China;

*These authors contributed equally to this work.

#Correspondence to:

Professor. Peng Liang,
Department of Neurosurgery, Harbin Medical University Cancer hospital, No.150, Haping Road, Nangang District, Harbin 150001, Heilongjiang, China.
TEL/Fax:+86-451-85718215. E-mail: liangpengd@yahoo.com
Professor. Xin Gao,
Computational Bioscience Research Center, King Abdullah University of Science and Technology (KAUST), Thuwal, 23955, Saudi Arabia. E-mail: xin.gao@kaust.edu.sa

*These authors contributed equally to this work.

Authors' contributions

C. L., H. Z., Y. H., H. J. and J. X. designed and performed the experiments, analyzed and interpreted data, and C. L. and H. L. wrote the manuscript; Y. .H., S. A. and Y. W. provided technical and material support, and T. S. , H. J. and G. S. acquisition and analyzed data; X. Xiao, T. F. and Y. W. edited the manuscript; P. L. and G. X. designed experiments and critically reviewed the paper.

Yours sincerely,

Chenlong Li

First Author:

Chenlong Li, Hongshan Zheng,

Jinsheng Xiong,

Yuxin Huang, Haoyang Li ,

Hua Jin, Hua Jin
Siqi Ai, Siqi Ai

Yingjie Wang, Yingjie Wang
Tianqi Su, Tianqi Su

Guiyin Sun, Guiyin. Sun
Xu Xiao, Xu Xiao

Tianjiao Fu, [illegible] Fu
Yujie Wang, Yujie Wang

Tianjiao Fu

Corresponding author

Xin Gao, Xin GAO
Peng Liang Peng Liang
